# Colchicine treatment in amyotrophic lateral sclerosis: safety, biological and clinical effects in a randomized clinical trial

**DOI:** 10.1093/braincomms/fcae304

**Published:** 2024-09-05

**Authors:** Giulia Gianferrari, Riccardo Cuoghi Costantini, Valeria Crippa, Serena Carra, Valentina Bonetto, Orietta Pansarasa, Cristina Cereda, Elisabetta Zucchi, Ilaria Martinelli, Cecilia Simonini, Roberto Vicini, Nicola Fini, Francesca Trojsi, Carla Passaniti, Nicola Ticozzi, Alberto Doretti, Luca Diamanti, Giuseppe Fiamingo, Amelia Conte, Eleonora Dalla Bella, Eustachio D’Errico, Eveljn Scarian, Laura Pasetto, Francesco Antoniani, Veronica Galli, Elena Casarotto, Co-ALS Investigators Group, Jessica Mandrioli, Nicola Fini, Ilaria Martinelli, Elisabetta Zucchi, Giulia Gianferrari, Cecilia Simonini, Francesca Prompicai, Silvia Parisi, Roberto D’Amico, Federico Banchelli, Roberto Vicini, Riccardo Cuoghi Costantini, Angelo Poletti, Valeria Crippa, Elena Casarotto, Serena Carra, Laura Mediani, Francesco Antoniani, Veronica Galli, Valentina Bonetto, Laura Pasetto, Orietta Pansarasa, Eveljn Scarian, Cristina Cereda, Francesca Trojsi, Carla Passaniti, Vincenzo Silani, Nicola Ticozzi, Alberto Doretti, Luca Diamanti, Giuseppe Fiamingo, Mario Sabatelli, Amelia Conte, Giulia Bisogni, Giuseppe Lauria, Eleonora Dalla Bella, Nilo Riva, Enrica Bersano, Isabella Laura Simone, Eustachio D’Errico, Roberto D’Amico, Angelo Poletti, Jessica Mandrioli

**Affiliations:** Department of Biomedical, Metabolic and Neural Sciences, University of Modena and Reggio Emilia, Modena 41121, Italy; Department of Neurosciences, Azienda Ospedaliero Universitaria di Modena, Modena 41126, Italy; Unit of Statistical and Methodological Support to Clinical Research, Azienda Ospedaliero-Universitaria, Modena 41121, Italy; Dipartimento di Scienze Farmacologiche e Biomolecolari ‘Rodolfo Paoletti’, Università degli Studi di Milano, Milan 20122, Italy; Dipartimento di Eccellenza 2018-2027, Università degli Studi di Milano, Milan 20122, Italy; Department of Biomedical, Metabolic and Neural Sciences, University of Modena and Reggio Emilia, Modena 41121, Italy; Research Center for ALS, Istituto di Ricerche Farmacologiche Mario Negri IRCCS, Milan 20156, Italy; Cellular Model and Neuroepigenetics Unit, IRCCS Mondino Foundation, Pavia 27100, Italy; Department of Pediatrics, Center of Functional Genomics and Rare diseases, ‘V. Buzzi’ Children’s Hospital, Milan 20154, Italy; Department of Neurosciences, Azienda Ospedaliero Universitaria di Modena, Modena 41126, Italy; Neurosciences PhD Program, University of Modena and Reggio Emilia, Modena 41121, Italy; Department of Neurosciences, Azienda Ospedaliero Universitaria di Modena, Modena 41126, Italy; Clinical and Experimental Medicine PhD Program, University of Modena and Reggio Emilia, Modena 41121, Italy; Department of Neurosciences, Azienda Ospedaliero Universitaria di Modena, Modena 41126, Italy; Unit of Statistical and Methodological Support to Clinical Research, Azienda Ospedaliero-Universitaria, Modena 41121, Italy; Department of Neurosciences, Azienda Ospedaliero Universitaria di Modena, Modena 41126, Italy; Department of Advanced Medical and Surgical Sciences, ALS Center, Università degli Studi della Campania L. Vanvitelli, Naples 80138, Italy; Department of Advanced Medical and Surgical Sciences, ALS Center, Università degli Studi della Campania L. Vanvitelli, Naples 80138, Italy; Department of Neurology, IRCCS Istituto Auxologico Italiano, Milan 20149, Italy; Department of Pathophysiology and Transplantation, ‘Dino Ferrari’ Center, Università degli Studi di Milano, Milan 20122, Italy; Department of Neurology, IRCCS Istituto Auxologico Italiano, Milan 20149, Italy; Neuroncology and Neuroinflammation Unit, IRCCS Mondino Foundation, Pavia 27100, Italy; Neuroncology and Neuroinflammation Unit, IRCCS Mondino Foundation, Pavia 27100, Italy; Department of Aging, Neurological, Orthopedic and Head-Neck Sciences, Adult NEMO Clinical Center, Unit of Neurology, Fondazione Policlinico Universitario A. Gemelli IRCCS, Rome 00168, Italy; 3rd Neurology Unit and Motor Neuron Disease Centre, Fondazione IRCCS Istituto Neurologico Carlo Besta, Milan 20133, Italy; Department of Basic Medical Sciences, ALS Center, Neurosciences and Sense Organs, University of Bari, Bari 70124, Italy; Cellular Model and Neuroepigenetics Unit, IRCCS Mondino Foundation, Pavia 27100, Italy; Research Center for ALS, Istituto di Ricerche Farmacologiche Mario Negri IRCCS, Milan 20156, Italy; Department of Biomedical, Metabolic and Neural Sciences, University of Modena and Reggio Emilia, Modena 41121, Italy; Department of Biomedical, Metabolic and Neural Sciences, University of Modena and Reggio Emilia, Modena 41121, Italy; Dipartimento di Scienze Farmacologiche e Biomolecolari ‘Rodolfo Paoletti’, Università degli Studi di Milano, Milan 20122, Italy; Dipartimento di Eccellenza 2018-2027, Università degli Studi di Milano, Milan 20122, Italy; Unit of Statistical and Methodological Support to Clinical Research, Azienda Ospedaliero-Universitaria, Modena 41121, Italy; Department of Medical and Surgical Sciences for Children and Adults, University of Modena and Reggio Emilia, Modena 41124, Italy; Dipartimento di Scienze Farmacologiche e Biomolecolari ‘Rodolfo Paoletti’, Università degli Studi di Milano, Milan 20122, Italy; Dipartimento di Eccellenza 2018-2027, Università degli Studi di Milano, Milan 20122, Italy; Department of Biomedical, Metabolic and Neural Sciences, University of Modena and Reggio Emilia, Modena 41121, Italy; Department of Neurosciences, Azienda Ospedaliero Universitaria di Modena, Modena 41126, Italy

**Keywords:** amyotrophic lateral sclerosis, colchicine, randomized clinical trial, protein quality control, neuroinflammation

## Abstract

In preclinical studies, the anti-inflammatory drug colchicine, which has never been tested in amyotrophic lateral sclerosis, enhanced the expression of autophagy factors and inhibited accumulation of transactive response DNA-binding protein 43 kDa, a known histopathological marker of amyotrophic lateral sclerosis. This multicentre, randomized, double-blind trial enrolled patients with probable or definite amyotrophic lateral sclerosis who experienced symptom onset within the past 18 months. Patients were randomly assigned in a 1:1:1 ratio to receive colchicine at a dose of 0.005 mg/kg/day, 0.01 mg/kg/day or placebo for a treatment period of 30 weeks. The number of positive responders, defined as patients with a decrease lesser than 4 points in the Amyotrophic Lateral Sclerosis Functional Rating Scale-Revised total score during the 30-week treatment period, was the primary outcome. Disease progression, survival, safety and quality of life at the end of treatment were the secondary clinical outcomes. Secondary biological outcomes included changes from baseline to treatment end of stress granule and autophagy responses, transactive response DNA-binding protein 43 kDa, neurofilament accumulation and extracellular vesicle secretion, between the colchicine and placebo groups. Fifty-four patients were randomized to receive colchicine (*n* = 18 for each colchicine arm) or placebo (*n* = 18). The number of positive responders did not differ between the placebo and colchicine groups: 2 out of 18 patients (11.1%) in the placebo group, 5 out of 18 patients (27.8%) in the colchicine 0.005 mg/kg/day group (odds ratio = 3.1, 97.5% confidence interval 0.4–37.2, *P* = 0.22) and 1 out of 18 patients (5.6%) in the colchicine 0.01 mg/kg/day group (odds ratio = 0.5, 97.5% confidence interval 0.01–10.2, *P* = 0.55). During treatment, a slower Amyotrophic Lateral Sclerosis Functional Rating Scale-Revised decline was detected in patients receiving colchicine 0.005 mg/kg/day (mean difference = 0.53, 97.5% confidence interval 0.07–0.99, *P* = 0.011). Eight patients experienced adverse events in placebo arm (44.4%), three in colchicine 0.005 mg/kg/day (16.7%) and seven in colchicine 0.01 mg/kg/day arm (35.9%). The differences in adverse events were not statistically significant. In conclusion, colchicine treatment was safe for amyotrophic lateral sclerosis patients. Further studies are required to better understand mechanisms of action and clinical effects of colchicine in this condition.

## Teaser

Gianferrari *et al*. report that colchicine treatment in amyotrophic lateral sclerosis patients was safe but did not meet the primary outcome in a phase 2 clinical trial. Although the lower-dose group showed a slower disease progression, further studies are recommended to understand colchicine’s mechanisms and effects in amyotrophic lateral sclerosis.

## Graphical Abstract

**Graphical Abstract:**
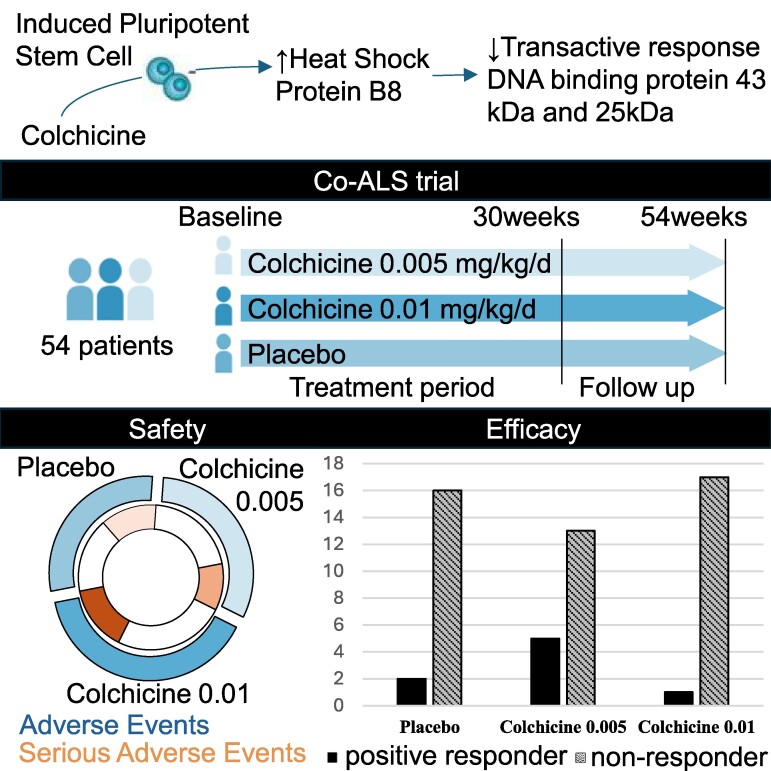

## Introduction

Amyotrophic lateral sclerosis is a devastating neurodegenerative disease that results in progressive disability and death usually in 3–5 years from symptom onset.^1^ The presence of misfolded proteins prone to aggregation and altered protein quality control, which leads to protein accumulation, thereby altering several intracellular functions,^2^ is a common feature in animal models and amyotrophic lateral sclerosis patients.

Detecting and promoting the removal of the misfolded protein in amyotrophic lateral sclerosis patient^3-5^ is mediated by the heat shock protein B8 (HSPB8), which works with the co-chaperone BAG3, forming the HSPB8–BAG3–HSP70 complex.^6^ More specifically, HSPB8 inhibits TDP-43 and its C-terminal fragment of 25 kDa (TDP-25) accumulation. TDP-25 contains a prion-like domain and is highly prone to aggregation.^3,4^ Finally, the HSPB8–BAG3–HSP70 complex upholds ‘granulostasis’, a surveillance mechanism that prevents dynamic stress granules (SGs) from converting into assemblies prone to aggregation.^7,8^

Colchicine is capable of significantly increasing HSPB8 expression and counteracting the accumulation of misfolded TDP-43 and TDP-25 species, as demonstrated in our previous publication using high-throughput screening in neuronal cells.^9^

Colchicine is an affordable medication with strong anti-inflammatory properties. It is approved to treat gout and familial Mediterranean fever, as well as other diseases, including Behçet’s disease, pericarditis and primary biliary cirrhosis. Its effects on leucocyte adhesion, migration, cytokine production and secretion are the better-recognized mechanisms of action.^10^ Intracellular assembly of the inflammasome complex in neutrophils and monocytes is disrupted by colchicine, inhibiting the nucleotide-binding domain, leucine-rich-containing family, pyrin domain-containing-3 and reducing serum levels of functional IL-1b and IL-2.^11^

Besides its anti-inflammatory properties, colchicine enhances autophagy by upregulating the master regulator transcription factor EB (TFEB), the adaptor protein SQSTM1/p62 and the autophagy player microtubule-associated protein 1A/1B-light chain 3 (MAP1LC3).^9^ Furthermore, colchicine stimulates HSPB8 expression independently of TFEB. The concomitant stimulation of HSPB8 and TFEB by colchicine may promote autophagic clearance of misfolded TDP-43 and its 25 kDa fragment, whose accumulation is involved in both sporadic and familial amyotrophic lateral sclerosis.^9,12^

The results of a phase 2, multicentre, randomized, double-blind, placebo-controlled clinical trial, Co-ALS, evaluating the effects of oral colchicine in individuals with amyotrophic lateral sclerosis are reported here.^13^

## Materials and methods

### Study design

Between 2018 and 2021, a randomized, double-blind, placebo-controlled clinical trial was conducted across seven amyotrophic lateral sclerosis referral centres in Italy. The study adhered to the Good Clinical Practice guidelines set by the International Council for Harmonization of Technical Requirements for Pharmaceuticals (ICH) and followed the ethical principles outlined in the Declaration of Helsinki.

Ethical Committee and Agenzia Italiana del Farmaco (AIFA) approved the study on 8 August 2018 and 19 September 2018, respectively. It was registered on 3 October 2018, the first patient was enrolled on 10 April 2019, and the last exit the study on 3 November 2021 (ClinicalTrials.gov, NCT03693781; https://www.clinicaltrialsregister.eu/ctr-search/trial/2017-004459-21/IT). Prior to screening, all patients granted a written informed consent. The trial, funded by AIFA through the ‘Bando per la ricerca indipendente 2016’, was conducted as a non-profit study.

The Azienda Ospedaliero Universitaria (AOU) of Modena served as the coordinating centre for the trial. The steering committee, consisting of the local principal investigators, collaboratively contributed to the design of the study, data analysis, drafting and submission of the manuscript (Supplementary Appendix, Section 1).

Data collected at each centre were entered into an online case report form (eCRF) provided by AOU of Modena.

At the beginning of the trial, an independent data and safety monitoring board was formed to regularly review unblinded safety data (Acknowledgements section). The Unit of Statistical and Methodological Support to Clinical Research, AOU of Modena, Italy, performed statistical analyses. The authors affirm their adherence to the Co-ALS study protocol, data completeness and accuracy.^13^

### Trial participants

Adult amyotrophic lateral sclerosis patients aged 80 years or younger, who experienced symptom onset within 18 months prior to screening, were included in the study. Inclusion criteria required participants to have a body mass index greater than 18, a body weight exceeding 50 kg and a forced vital capacity greater than 65%. Additionally, all participants had to maintain a stable riluzole dose for a minimum of 30 days before screening. Patients diagnosed with possible amyotrophic lateral sclerosis as defined by the Revised El Escorial criteria were exluded.^14^ Only classic or bulbar phenotypes could be enrolled, without known pathogenic mutations in *SOD1*, *TARDBP*, *FUS* and *C9ORF72*, to reduce clinical heterogeneity. Exclusion criteria covered diseases and conditions that would contraindicate colchicine, including food or co-medications strongly inhibiting cytochrome P450 3A4, and/or the presence of severe/advanced comorbidities. Patients receiving chronic treatment with anti-inflammatory/immunosuppressive/immunomodulating drugs were excluded. Use of highly effective contraception was required.^15^ The published protocol details the inclusion and exclusion criteria.^13^

### Randomization and masking

Eligible patients were randomly assigned in a 1:1:1 ratio to one of the three treatment groups: colchicine 0.01 mg/kg of body weight a day (mg/kg/day) (18 patients), colchicine 0.005 mg/kg/day (18 patients) or placebo (18 patients). The randomization was conducted using a permuted block design with blocks of three and six. An unblinded statistician generated the randomization schedule by Stata Statistical Software (Release 15. College Station, TX: StataCorp LLC). The rate of disease progression at screening from the onset of symptoms, with a threshold set at </≥0.7 was used for computerized randomization stratification. The progression rate at randomization was measured by assessing the monthly decrease of Amyotrophic Lateral Sclerosis Functional Rating Scale-Revised (ALSFRS-R) score, considering an initial score of 48 at symptom onset.

Each patient was uniquely identified by a code that stayed consistent throughout the duration of the trial. An authorized company distributed trial drug in kits labelled with randomly generated four-digit numeric codes (STM Pharma Pro S.R.L, Grezzago, Milan, Italy; https://stmpharmapro.it/en/). When a new patient was recruited, kits were sent sequentially to the sites. STM Pharma Pro S.R.L guaranteed that the active treatment and placebo were identical to both investigators and participants, in full compliance with the Good Manufacturing Practices of the European Union for active pharmaceutical ingredients and ICH Q7A guidelines.

The investigator received technical tools and password details to selectively unlock the code for a specific patient in case of emergency where knowing the administered drug was essential for proper treatment. If this occurred, the patient would have been withdrawn from the study (this never happened).

### Procedures

The investigational medical product was prepared by STM Pharma Pro S.R.L. in adherence to Good Manufacturing Procedure and Good Clinical Practice guidelines, and national legal requirements, as previously reported.^13^ Procedures for the storage, dispensation, return and destruction of the investigational medical product have been detailed in the previously published protocol.^13^ Logistics were managed by STM Pharma Pro S.R.L.

Treatment was administered orally, at fast, twice a day. The daily dosage of colchicine could range from 0 (placebo group) to 1 mg (0.01 mg/kg/day arm and body weight >70 kg) depending on the body weight and treatment arm. The treatment period lasted 30 weeks, followed by a 24-week observation phase. Every 28 days, patients received four blisters, each containing 15 tablets of either the active medication or placebo, based on the designated treatment group. Caring neurologist could request a dose reduction via eCRF if adverse events (AEs) or reactions were suspected to be related to the study drug, on clinical judgment. The protocol permitted multiple steps for dose reduction as needed.

### Outcomes

The proportion of patients exhibiting a positive response in amyotrophic lateral sclerosis progression (considered as a decrease in ALSFRS-R total score fewer than 4 points in 30 weeks) comparing baseline (T0) and treatment end (Week 30 or T1) between colchicine and placebo arms was the primary outcome.

The ALSFRS-R is a widely used clinical scale for assessing the functional status of amyotrophic lateral sclerosis patients. It consists of 12 questions that evaluate bulbar, fine motor, gross motor and respiratory domains. Each item is scored from 4 (full function) to 0 (complete impairment) resulting in a total score ranging from 48, corresponding to full retention of all 4 functions, to 0, reflecting a total loss of these functions.^16^

Secondary endpoints consisted of the following:

- Safety profile and tolerance of colchicine treatment assessed by analysing any AEs and monitoring alterations in clinical and laboratory examinations. Symptoms indicative of disease advancement were documented as AEs.
- Evaluation of biological activity by measuring changes from baseline to Week 30 (in blood mononuclear cells and fibroblasts) and Week 54 (in blood mononuclear cells) comparing colchicine and placebo arms, of the following: (i) the levels of mRNA and proteins such as SQSTM1/p62, TFEB, MAP1LC3B, autophagy related genes, BAG1, BAG3, HSPB8, the inducible HSP70 family member 6 (HSPA6) and HSF1; (ii) SG response; (iii) the levels and relative proportion of soluble and insoluble forms of TDP-43, TDP-43 fragments, ubiquilin 2 (UBQLN), SQSTM1/p62 and optineurin (OPTN); and (iv) changes in RNA profile. Furthermore, we compared the change from baseline to Week 30 in the colchicine and placebo groups of (i) extracellular vesicle secretion with analysis of their content of hyperphosphorylated TDP-43, SQSTM1/p62, UBQLN and OPTN; (ii) plasma creatinine, albumin, creatine kinase (CK) and vitamin D; and (iii) selected markers of neurodegeneration and inflammation, including plasma/CSF neurofilaments, MCP1, IL17, IL18 and IL-18BP.
- Comparison of clinical endpoints between the colchicine arms and placebo: (i) tracheostomy-free survival from baseline and survival rates at treatment and study end and (ii) forced vital capacity scores, expressed as the percentage of the predicted normal value, adjusted for age, sex, weight and height, from baseline to Weeks 8, 18, 30, 42 and 54. The highest score from three trials was used for analysis.
- Changes from baseline to Weeks 8, 30 and 54 in ALSAQ-40, a 40-item, amyotrophic lateral sclerosis–specific quality of life scale. It includes five dimensions corresponding to eating and drinking, communication, activities of daily living/independence, mobility and emotional well-being. Patients complete the questionnaire based on their experiences over the past 2 weeks, rating them on a 5-point Likert scale. The responses are then converted into a summary score, with 0 indicating the best health status and100 representing the worst.^17^

Details on laboratory procedures and clinical outcome measures are reported in Supplementary Appendix, Section 2.

### Statistical analysis

Sample size was calculated by evaluating the proportion of participants without significant disease progression at 30 weeks (end of treatment) in the colchicine-treated versus placebo group. Disease progression was evaluated using the ALSFRS-R, whose monthly decline has been described as 0.89 ± 0.13 points/month.^18^ Considering a treatment duration of 30 weeks, most amyotrophic lateral sclerosis patients would have an ALSFRS-R decline of 6.23 after 30 weeks, with 90% of patients having a decline between 4.7 and 7.7. We expected that only 10% of amyotrophic lateral sclerosis patients in the placebo arm would not have an ALSFRS-R decrease of at least 4 points (a difference considered clinically significant^19^) from baseline, while a significant number of those receiving treatment, quantified up to 60%, would not have had the same decline.

By randomly assigning participants in a 1:1:1 ratio across the three arms, we calculated that enrolling 51 amyotrophic lateral sclerosis patients would achieve 80% power to identify a decline of fewer than 4 points in the ALSFRS-R total score in at least 60% of those receiving treatment, compared to under 10% in the placebo arm, using a *χ*^2^ test with a two-sided alpha level of 0.025 and no corrections applied.

To account for a potential 5% dropout rate, the study was designated to enrol 54 patients.

All participants who took at least one dose of investigational medical product were included in safety analyses.

At each visit, all AEs, severe AEs (SAEs) and those resulting in withdrawal from treatment were documented in accordance with to ICH guidelines. These events were catalogued and compared across the different treatment groups.

All the patients who assumed at least one tablet of the experimental treatment were considered in the intention-to-treat analysis.

The per protocol analysis excluded patients assuming the drug with major protocol deviations (i.e. participants who adhered to less than 80% of the prescribed therapy).

To compare the colchicine and placebo arms, mean and standard deviation (SD) or median and interquartile range were applied to continuous variables, while categorical variables were analysed using counts and percentages.

The primary outcome was analysed as the difference from baseline to treatment end in the proportion of participants showing a decrease in the ALSFRS-R global score of fewer than 4 points, between the placebo and colchicine groups. Univariable logistic regression models were applied to calculate the treatment arm’s effect. Results were reported as odds ratio (OR) with a 95% confidence interval (CI) when comparing the colchicine arm with the placebo arm. A 97.5% CI was applied when comparing each colchicine arm (colchicine 0.005 mg/kg/day and 0.01 mg/kg/day) to the placebo arm.

To evaluate changes from baseline to Week 30 and other intervals, we computed the mean absolute differences across treatment groups for various biomarkers, including indicators of autophagy, SG response and composition, soluble and insoluble forms of TDP-43, TDP-43 fragments, OPTN, UBQLN, SQSTM1/p62, extracellular vesicle secretion and neurofilaments. These comparisons were made using linear regression models, with treatment groups serving as the independent variables, and results were presented as mean differences (MDs) between the groups.

Associations between treatment arm and the difference in each biological parameter from baseline to Week 30 were also assessed using Wilcoxon–Mann–Whitney test. Pearson’s linear correlation coefficient was performed to test correlations between numerical variables.

The log-rank test was used to compare survival outcomes, including time to the initiation of permanent ventilation, tracheostomy or death.

A segmented repeated measures linear mixed model was employed to evaluate whether the mean monthly changes in selected numerical outcomes from baseline differed between treatment groups during three distinct time periods: pre-treatment (from symptom onset to baseline), during treatment (baseline to Week 30) and post-treatment (from Week 30 onwards).^20^ The raw outcome measurements were the dependent variables, while the independent variables included the treatment group, the interaction between the period (before, during or after treatment) and time (in months from baseline) and the interaction between treatment group, period and time.

To accommodate repeated observations within the same participants, a random intercept was included, and a random slope was added to address individual linear changes over time, as previously reported.^20^

The results were presented as the monthly outcome changes for the placebo group, alongside the MD in monthly changes when comparing the colchicine groups to the placebo. Both sets of results were displayed for the three time segments.

A *post hoc* analysis of tracheostomy-free survival with the last observation recorded on 3 November 2022 was also conducted.

The comparisons between the colchicine arms (0.005 mg/kg/day or 0.01 mg/kg/day) and the placebo group were adjusted for multiple arms comparison using the Bonferroni method. The Relative Risk (RR) was calculated to compare the probability of events between the treatment and placebo groups. RR is defined as the ratio of the probability of an event occurring in the treatment group compared to the control group. No correction was made for the overall comparison of colchicine versus placebo. The degree of uncertainty in the findings was reflected by the 95% CI when comparing the combined colchicine arms with the placebo arm. A 97.5% CI was applied when comparing each colchicine arm (colchicine 0.005 mg/kg/day and 0.01 mg/kg/day) to the placebo group. A *P* value of 0.05 or less was deemed statistically significant for the overall colchicine versus placebo comparison, while a *P* value of 0.025 or less was considered significant for the individual comparisons of each colchicine dosage to the placebo group.

Analyses were performed using Stata software, version 15 (StataCorp. 2017. Stata Statistical Software: Release 15. College Station, TX: StataCorp LLC) and R software, version 4.1.1 (The R Foundation for Statistical Computing, Wien).

### Ethics considerations

The study received ethical committee approval on 19 September 2018 (Comitato etico Area Vasta Emilia Nord, file number 366/2018/FARM/AOUMO). The study was approved by Italian Drug Regulatory Agency (AIFA) on 8 August 2018.

All the participants provided written informed consent before screening.

## Results

**Figure 1:**
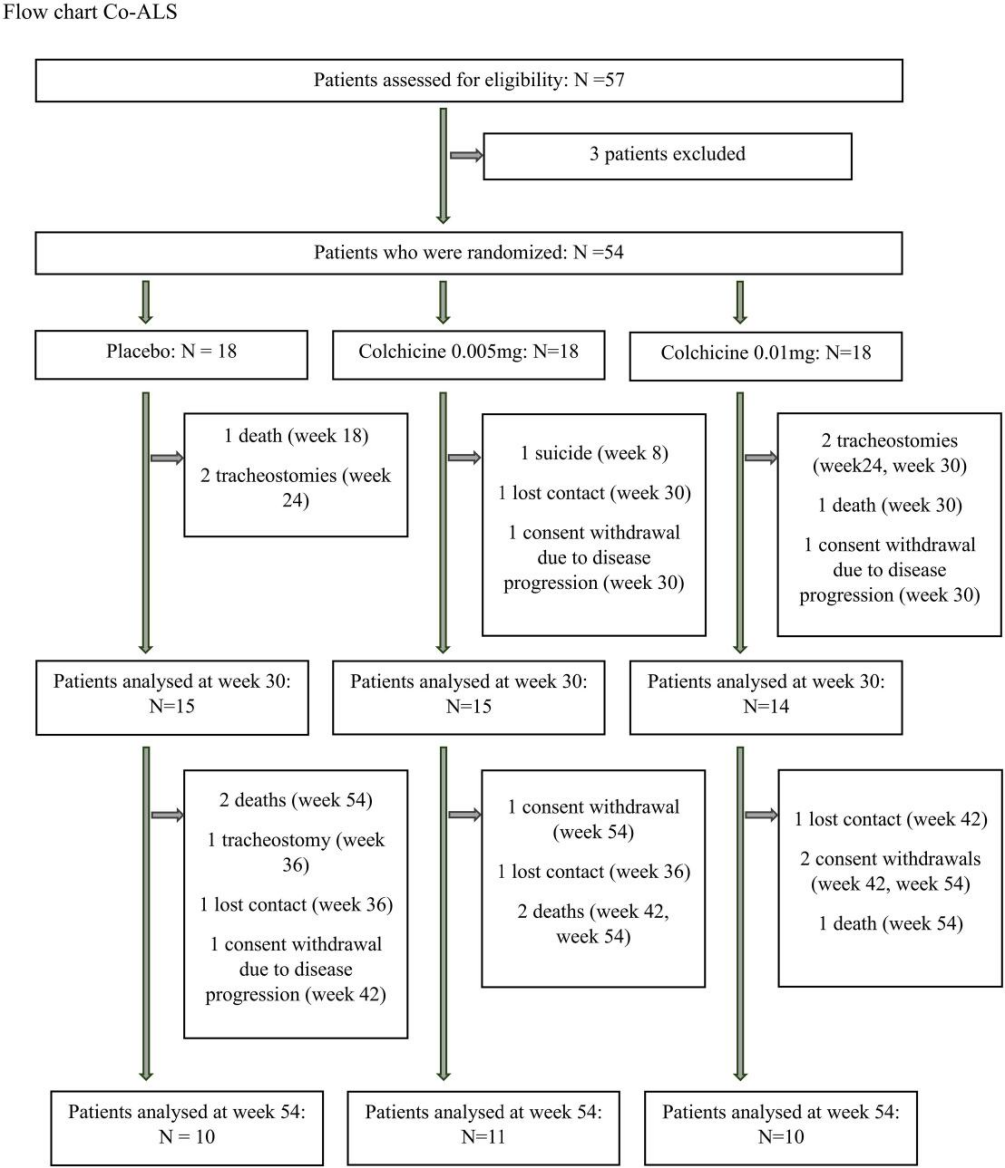
**CONSORT diagram of the study reporting screening, randomization and follow-up of amyotrophic lateral sclerosis patients enrolled in the trial.** Time inside brackets indicate the first week in which the outcome was missing.

Of the 57 persons with amyotrophic lateral sclerosis screened for eligibility, 54 were randomly assigned to a trial group: 18 to colchicine 0.005 mg/kg/day, 18 to colchicine 0.01 mg/kg/day and 18 to placebo (Fig. 1).

For 10 patients, the primary outcome measure at Week 30 could not be calculated due to death/tracheostomy (6 individuals), suicide (1), withdrawal of patient consent due to disease progression (2) and loss of contact (1). Twenty-three patients did not complete the follow-up until 54 weeks. No patient took less than 80% of the study drug as planned per protocol; therefore, both the per protocol and intention-to-treat analyses yielded the same results.

Table 1 summarizes baseline demographic and disease features of the trial participants. There were not unbalanced factors among treatment arms. Baseline biological features are summarized in Supplementary Tables 1–3.

**Table 1:** Demographic and clinical characteristics of the participants at baseline (ITT population)

Characteristic	Placebo (*N* = 18)	Colchicine 0.005 mg/kg/day (*n* = 18)	Colchicine 0.01 mg/kg/day (*n* = 18)
Male sex, *n* (%)	13 (72.2)	13 (72.2)	10 (55.5)
Age, years	57.33 ± 9.36	56.61 ± 12.61	58.11 ± 11.67
Bulbar onset, *n* (%)	4 (22.2)	3 (16.7)	5 (27.8)
Upper limb onset, *n* (%)	7 (38.9)	8 (44.4)	5 (27.8)
Lower limb onset, *n* (%)	7 (38.9)	7 (38.9)	8 (44.4)
Months since amyotrophic lateral sclerosis symptom onset	11.94 ± 4.86	12.72 ± 5.34	12.39 ± 4.80
ALSFRS-R total score	40.78 ± 4.54	39.39 ± 5.44	39.00 ± 5.42
Bulbar score	11.11 ± 1.53	10.00 ± 2.25	10.33 ± 2.17
Fine-motor score	8.89 ± 2.22	8.44 ± 2.99	8.50 ± 3.45
Gross-motor score	8.89 ± 2.83	9.06 ± 2.86	8.39 ± 2.75
Breathing score	11.89 ± 0.47	11.89 ± 0.47	11.78 ± 0.94
Pre-baseline ALSFRS-R slope^a^	0.84 ± 0.51	0.63 ± 0.35	0.68 ± 0.41
Pre-baseline ALSFRS-R slope^b^	0.62 (0.34, 0.98)	0.57 (0.42, 0.88)	0.70 (0.45, 1.05)
Forced vital capacity, % of predicted normal value	95.78 ± 16.42	98.17 ± 16.02	97.61 ± 22.53
Body mass index, kg/m^2^	24.76 ± 3.60	24.53 ± 2.90	25.35 ± 2.60
ALSAQ40 total score	48.06 ± 26.73	51.28 ± 31.02	60.50 ± 25.08

ITT, intention to treat; ALSFRS-R, Amyotrophic Lateral Sclerosis Functional Rating Scale-Revised; ALSAQ40, Amyotrophic Lateral Sclerosis Assessment Questionnaire.

^a^Pre-baseline ALSFRS-R slope has been calculated as monthly decline of ALSFRS-R score assuming a total score of 48 at onset and expressed as means and standard deviations.

^b^Pre-baseline ALSFRS-R slope has been calculated as monthly decline of ALSFRS-R score assuming a total score of 48 at onset and expressed as medians and interquartile ranges.

A total of 20 out of 36 individuals (55.6%) in the colchicine groups and 9 out of 18 individuals (50%) in the placebo group experienced one or more AEs throughout the study (RR = 1.11, 95% CI 0.64–1.91, *P* = 0.700); individuals with AEs were 13 out of 18 (72.1%) in the colchicine 0.01 mg/kg/day group and 7 out of 18 (38.9%) in the colchicine 0.005 mg/kg/day group, both not statistically different from the placebo group (RR = 1.44, 97.5% CI 0.84–2.49, *P* = 0.171, and RR = 0.78, 97.5% CI 0.37–1.63, *P* = 0.502, respectively) (Supplementary Table 4).

Individuals with SAEs were 6 out of 18 (33.3%) in the placebo group and 12 out of 36 (33.3%) in the colchicine groups (RR = 1.00, 95% CI 0.45–2.23, *P* = 1.00) (Supplementary Table 5).

The total number of AEs was 14 for the placebo arm and 34 for the colchicine arms. SAEs were 6 in the placebo group (42.8% of total AEs in this group) and 12 in the colchicine groups (35.3% of total AEs in these groups) (Supplementary Table 6).

Among the totality of AEs, only two caused treatment discontinuation, both in the colchicine groups (Supplementary Table 6).

Events that were slightly more common in the colchicine group were primarily accidents and injuries, followed by respiratory disorders, metabolic and nutritional disorders, pruritus, depression and self-injury and cardiac disorders (Table 2).

**Table 2:** Treatment-emergent adverse events classified according to MedDRA dictionary

	Placebo (*n* = 18)		Colchicine 0.005 mg/kg/day (*n* = 18)		Colchicine 0.01 mg/kg/day (*n* = 18)	
Adverse events MedDRA preferred term	*n*	%	*n*	%	*n*	%
Gastrointestinal non-specific dysfunction	1	5.5%	0	0.0%	2	11.1%
Gastro-oesophageal reflux disease	1	5.5%	0	0.0%	1	5.5%
Diarrhoea^a^	0	0.0%	0	0.0%	1	5.5%
Depression and suicide/self-injury	0	0.0%	1	5.5%	0	0.0%
Suicide/self-injury	0	0.0%	1	5.5%	0	0.0%
Infections and infestations	1	5.5%	0	0.0%	0	0.0%
Sinusitis	1	0.0%	0	0.0%	0	0.0%
Accidents and injuries	3	16.7%	4	22.2%	5	27.8%
Fall	3	16.7%	2	11.1%	3	16.7%
Head injury	0	0.0%	0	0.0%	1	5.5%
Foot fracture	0	0.0%	0	0.0%	1	5.5%
Wrist fracture	0	0.0%	1	5.5%	0	0.0%
Hand fracture	0	0.0%	1	5.5%	0	0.0%
Investigation	0	0.0%	1	5.5%	0	0.0%
Abnormal blood tests^a^	0	0.0%	1	5.5%	0	0.0%
Metabolism and nutrition disorders	0	0.0%	1	5.5%	3	16.7%
Dysphagia	0	0.0%	1	5.5%	3	16.7%
Musculoskeletal and connective tissue disorders	1	5.5%	0	0.0%	0	0.0%
Lumbo-sacral pain	1	5.5%	0	0.0%	0	0.0%
Respiratory, thoracic and mediastinal disorders	7	38.9%	6	33.3%	9	50.0%
Bronchitis	0	0.0%	1	5.5%	1	5.5%
Pneumonia	1	5.5%	3	16.7%	3	16.7%
Pneumonia—pulmonary embolism	1	5.5%	0	0.0%	0	0.0%
Respiratory failure	5	27.8%	2	11.1%	5	27.8%
Skin and subcutaneous tissue disorders	0	0.0%	1	5.5%	0	0.0%
Pruritus	0	0.0%	1	5.5%	0	0.0%
Vascular disorders	1	5.5%	0	0.0%	0	0.0%
Embolic and thrombotic events. venous	1	5.5%	0	0.0%	0	0.0%
Cardiac disorders	0	0.0%	1	5.5%	0	0.0%
Cardiac failure	0	0.0%	1	5.5%	0	0.0%

MedDRA, Medical Dictionary for Regulatory Activities.

^a^These events were considered as possibly related to the study drug.

Most SAEs were complications related to disease progression, such as dysphagia and hospitalization for gastrostomy, or respiratory failure/pneumonia. Among the eight deaths that occurred during the study, five were due to respiratory insufficiency related to disease progression, two patients died from cardiac arrest and one subject committed suicide (Supplementary Table 7).

One subject in the colchicine 0.01 mg/kg/day treatment arm experienced dysentery and vomiting, probably related to the study drug. There were no reported permanent consequences from this event.

Among the patients who received colchicine treatment, 6 out of 36 (16.7%) experienced a decline in ALSFRS-R score of fewer than 4 points within 30 weeks, compared to 2 out of 18 (11.1%) in the placebo group (OR = 1.60, 95% CI 0.24–18.4, *P* = 0.552). Compared to the placebo group, a positive response was observed in 5 out of 18 (27.8%) patients treated with colchicine at a dose of 0.005 mg/kg/day (OR = 3.08, 97.5% CI 0.39–37.2, *P* = 0.208) and in 1 out of 18 (5.6%) patients treated with colchicine at a dose of 0.01 mg/kg/day (OR = 0.47, 97.5% CI 0.01–10.2, *P* = 0.589) (Table 3).

**Table 3:** Patients exhibiting a positive response (ALSFRS-R decline < 4 points in 30 weeks), comparing baseline and treatment end between colchicine and placebo arm

Primary outcome	Placebo *N* = 18	Colchicine 0.005 mg/kg/day *N* = 18	Colchicine 0.01 mg/kg/day *N* = 18	Colchicine *N* = 36	Colchicine 0.005 mg/kg/day versus placebo	Colchicine 0.01 mg/kg/day versus placebo	Colchicine versus placebo
					Measure of association (97.5% CI)^a^	Measure of association (97.5% CI)^a^	Measure of association (95% CI)^a^
No. of patients with positive response (%)	2/15 (13.3)	5/15 (33.3)	1/14 (7.1)	6/29 (20.7)	OR = 3.25 (0.39–40.4)	OR = 0.50 (0.01–11.2)	OR = 1.70 (0.33–12.7)
No. of patients with positive response (%)^b^	2/18 (11.1)	5/18 (27.8)	1/18 (5.6)	6/36 (16.7)	OR = 3.08 (0.39–37.2)	OR = 0.47 (0.01–10.2)	OR = 1.60 (0.24–18.4)

ITT, intention to treat; OR, odds ratio; CI, confidence interval.

^a^Comparisons were carried out with logistic regression models. Comparison between colchicine 0.005 mg/kg/day or 0.01 mg/kg/day arms and the placebo arm was corrected using the Bonferroni method to account for multiple arm comparisons; therefore, CIs are set at 97.5% and *P* values are considered statistically significant if <0.025. We did not apply any correction to the comparison of colchicine versus placebo arm; therefore, CIs are set at 95% and *P* values are considered statistically significant if <0.05. ITT analysis.

^b^Patients who died, received tracheostomy, manifested disease progression, dropped out or were lost to follow-up were considered as non-positive responder.

At the end of treatment, the mean decrease of ALSFRS-R total score from baseline was 8.00 points in patients treated with colchicine (7.13 and 8.93 points among patients treated with colchicine 0.005 mg/kg/day and colchicine 0.01 mg/kg/day), compared to 9.67 in placebo patients (MD −1.67, 95% CI −5.03–1.70, *P* = 0.337, for colchicine arm; MD −2.53, 97.5% CI −6.96–1.89, *P* = 0.207, for colchicine 0.005 mg/kg/day arm; and MD −0.74, 97.5% CI −5.24–3.77, *P* = 0.715, for 0.01 mg/kg/day arm) (Supplementary Table 8).

While there were no differences in ALSFRS-R monthly variation between the colchicine and placebo arms before treatment, patients receiving colchicine 0.005 mg/kg/day had a slower decline during and after treatment compared to those who received the placebo (MD 0.53, 97.5% CI 0.07–0.99, *P* = 0.011, during treatment; MD 0.46, 97.5% CI 0.07–0.84, *P* = 0.010, after treatment). The slowing of disease progression was absent for the colchicine 0.01 mg/kg/day treatment group (MD −0.006, 97.5% CI −0.46–0.45, *P* = 0.976, during treatment; MD 0.00, 97.5% CI −0.38–0.38, *P* = 0.998, after treatment) and for the colchicine group as a whole (MD 0.26, 95% CI −0.09–0.61, *P* = 0.146, during treatment; MD 0.23, 95% CI −0.06–0.52, *P* = 0.127, after treatment) (Table 4). Supplementary Fig. 1 illustrates individual rate of decline in ALSFRS-R total score.

**Table 4:** Change in ALSFRS-R monthly decline before and during the treatment

			Monthly variation		Comparison with placebo			
Outcome	Period	Arm	Mean	Std. Err	MD	CI^a^		*P* value^a^
ALSFRS-R Total score	Before treatment	Placebo	−0.630	0.120				
	During treatment	Placebo	−1.625	0.146				
	Before treatment	Colchicine 0.005 mg/kg/day	−0.666	0.118	−0.035	−0.407	0.342	0.835
		Colchicine 0.01 mg/kg/day	−0.649	0.118	−0.018	−0.391	0.356	0.914
		Colchicine	−0.651	0.082	−0.025	−0.307	0.259	0.863
	During treatment	Colchicine 0.005 mg/kg/day	−1.090	0.146	0.535	0.075	0.991	**0**.**011**
		Colchicine 0.01 mg/kg/day	−1.631	0.145	−0.006	−0.462	0.450	0.976
		Colchicine	−1.366	0.103	0.262	−0.088	0.610	0.146

Average monthly variations before and during treatment for the placebo group, as well as the comparisons between arms, are shown. Comparisons were performed using segmented repeated measures linear mixed models. Three segments of time were analysed: before the treatment (from onset to baseline), during the treatment (after baseline and up to Week 30) and after treatment (from Week 30 to Week 54). The dependent variables were the raw measurements of the outcomes, whereas the independent variables were arm, time (months from baseline)×period (before or during treatment) interaction and arm × time × period interaction. A random intercept term was also used to account for repeated measurements over the same individual, and a random slope term was used to account for individual linear variations over time. Random slope terms were kept in the model if they improved the overall goodness of fit of the model. In Table 4, only results from two segments (before and during treatment) are reported. In bold are *P* values statistically significant.

ALSFRS-R, Amyotrophic Lateral Sclerosis Functional Rating Scale-Revised; MD, mean difference; Std. Err, standard error; CI, confidence interval.

^a^Comparison between colchicine 0.005 mg/kg/day or 0.01 mg/kg/day arms and the placebo arm was carried out using the Bonferroni method to account for multiple arm comparisons; therefore, CIs are set at 97.5% and *P* values are considered statistically significant if <0.025. We did not apply any correction to the comparison of colchicine versus placebo arm; therefore, CIs are set at 95% and *P* values are considered statistically significant if <0.05. ITT analysis.

The main secondary clinical outcomes are presented in Table 5.

**Table 5:** Main secondary clinical outcomes comparing baseline and treatment end between colchicine and placebo arm

Outcomes	Placebo *N* = 18	Colchicine 0.005 mg/kg/day *N* = 18	Colchicine 0.01 mg/kg/day *N* = 18	Colchicine *N* = 36	Colchicine 0.005 mg/kg/day versus placebo	Colchicine 0.01 mg/kg/day versus placebo	Colchicine versus placebo
					Measure of association (97.5% CI)^a^	Measure of association (97.5% CI)^a^	Measure of association (95% CI)^a^
No. deaths or tracheostomy	3/18 (16.7)	0	3/18 (16.7)	3/36(8.8)	Not estimable	HR = 0.93 (0.19–4.62)	HR = 0.47 (0.10–2.4)
Mean Δ_30wks-baseline_FVC (SD), *n*	−20.9 (18.6), 13	−11.7 (19.4), 9	−21.6 (17.4), 11	−17.2 (18.5), 20	MD = 9.3 (−8.7–27.2)	MD = −0.7 (−17.6–16.2)	MD = 3.8 (9.2–16.7)
Mean Δ_30wks-baseline_ALSAQ40 Total score (SD), *n*	15.2 (11), 15	8.8 (11.5), 15	13 (10.5), 14	10.8 (11), 29	MD = −6.4 (−15.4–2.6)	MD = −2.2 (−11.4–7.0)	MD = −4.4 (−12.3–3.5)
Mean monthly variation of ALSFRS-R total score (SE)	−1.63 (0.15)	−1.09 (0.15)	−1.63 (0.14)	−1.37 (0.10)	MD = 0.54 (0.08–0.99)	MD = −0.01 (−0.46–0.45)	MD = 0.26 (−0.09–0.61)

FVC, forced vital capacity; SD, standard deviation; SE, standard error; CI, confidence interval; Δ_30wks-baseline_, difference from baseline to Week 30; ITT, intention to treat.

^a^Comparisons were carried out with logistic regression models. Comparison between colchicine 0.005 mg/kg/day or 0.01 mg/kg/day arms and the placebo arm was corrected using the Bonferroni method to account for multiple arm comparisons; therefore, CIs are set at 97.5% and *P* values are considered statistically significant if <0.025. We did not apply any correction to the comparison of colchicine versus placebo arm; therefore, CIs are set at 95% and *P* values are considered statistically significant if <0.05. ITT analysis.

At the end of the study, eight patients had died, and five underwent tracheostomy, with no differences in tracheostomy-free survival from baseline among treatment arms (Supplementary Fig. 2; Supplementary Table 9).

**Figure 2:**
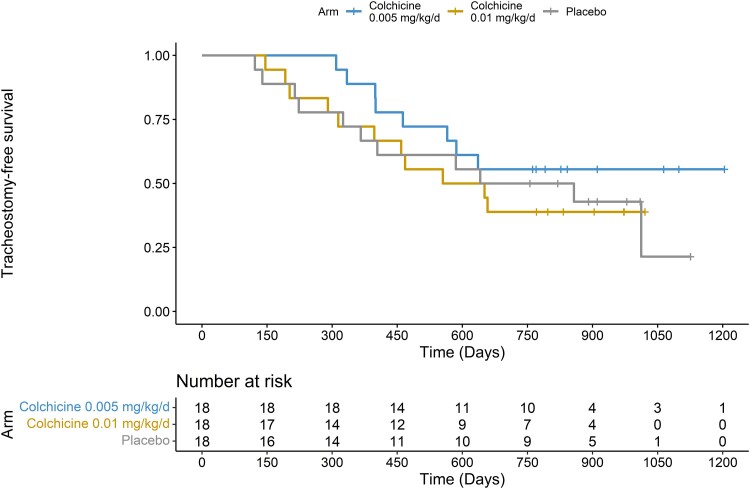
**Tracheostomy-free survival from baseline to 3 November 2022 (1 year after study end) based on treatment arm allocation.** Thick marks represent participants lost to follow-up. The number of participants at risk is displayed in the table.

A *post hoc* Cox regression analysis of tracheostomy-free survival with the last observation on 3 November 2022 (1 year after the last visit of the last patient) showed no significant differences among treatment arms (HR 0.63, 95% CI 0.25–1.60, *P* = 0.314, for colchicine 0.005 mg/kg/day; HR 1.06, 95% CI 0.46–2.45, *P* = 0.890, for colchicine 0.01 mg/kg/day) (Fig. 2).

Placebo and treatment arms showed no significant differences in respiratory function (Supplementary Table 10; Supplementary Fig. 3) and in the quality of life (Supplementary Table 11; Supplementary Fig. 4), even when considering clinical outcome measures monthly variations during and after treatment (Supplementary Table 12).

The main secondary biological outcomes are presented in Table 6.

**Table 6:** Main secondary biological outcomes comparing baseline and treatment end between colchicine and placebo arm

Biological outcomes	Placebo *N* = 18	Colchicine 0.005 mg/kg/day *N* = 18	Colchicine 0.01 mg/kg/day *N* = 18	Colchicine *N* = 36	Colchicine 0.005 mg/kg/day versus placebo	Colchicine 0.01 mg/kg/day versus placebo	Colchicine versus placebo
					*P* value^a^	*P* value^a^	*P* value^a^
Mean 2^−ΔΔCt^ HSPB1 (PBMC) (SD), *n*^b^	1.81 (1.38), 9	1.54 (0.87), 9	2.37 (2.21), 9	1.95 (1.69), 18	0.67	0.73	0.98
Mean 2^−ΔΔCt^ BAG3 (PBMC) (SD), *n*^b^	1.16 (0.46), 9	2.04 (1.31), 9	1.46 (1.25), 9	1.75 (1.27), 18	0.26	0.93	0.56
Mean 2^−ΔΔCt^ BAG1 (PBMC) (SD), *n*^b^	0.96 (0.83), 7	1.56 (1.04), 8	1.69 (1.39), 9	1.62 (1.2), 17	0.12	0.25	0.11
Mean 2^−ΔΔCt^ HSF1 (PBMC) (SD), *n*^b^	1.42 (0.77), 8	1.6 (1.13), 9	1.77 (1.35), 9	1.68 (1.21), 18	0.96	0.96	0.94
Mean 2^−ΔΔCt^ TFEB (PBMC) (SD), *n*^b^	1.33 (0.74), 9	1.77 (2.67), 9	2.4 (2.95), 9	2.08 (2.75), 18	0.55	1.00	0.71
Mean 2^−ΔΔCt^ SQSTM1.p62 (PBMC) (SD), *n*^b^	0.74 (0.34), 9	0.9 (0.45), 9	1.92 (1.59), 9	1.41 (1.25), 18	0.49	0.06	0.13
Mean 2^−ΔΔCt^ MAP1LC3B (PBMC) (SD), *n*^b^	0.88 (0.5), 9	1.1 (0.59), 9	2.46 (2.55), 9	1.78 (1.93), 18	0.61	0.08	0.18
Mean 2^−ΔΔCt^ HSPA6 (PBMC) (SD), *n*^b^	1.27 (1.09), 9	2.73 (2.95), 9	24.99 (36.6), 9	13.86 (27.67), 18	0.73	0.04	0.16
Mean Δ_30wks-baseline_TDP-43 (PBMC) (SD), *n*	1.68 (2.04), 4	−0.63 (0.33), 5	0.33 (1.25), 4	−0.21 (0.94), 9	0.03	0.18	**0**.**04**
Mean Δ_30wks-baseline_DRiP (fibroblasts) (SD), *n*	0.07 (0.33), 5	−0.06 (0.27), 3	0.04 (0.25), 7	0.00 (0.24), 10	0.857	0.91	0.81
Mean Δ_30wks-baseline_EXOs (plasma) (SD), *n*	0.12 (0.59), 7	−0.56 (1.35), 8	−0.34 (1.23), 8	−0.45 (1.26), 16	0.28	0.61	0.34
Mean Δ_30wks-baseline_MVs (plasma) (SD), *n*	0.09 (1.45), 7	−0.07 (0.58), 8	0.5 (1.2), 8	0.21 (0.95), 16	0.40	0.61	0.87
Mean Δ_30wks-baseline_NFL (plasma) (SD), *n*	3.8 (57.55), 14	−2.07 (17.33), 13	13.95 (30.15), 14	6.24 (25.68), 27	0.94	0.13	0.33
Mean Δ_30wks-baseline_IL17A (plasma) (SD), *n*	0.43 (1.12), 8	0.06 (0.11), 10	0.09 (0.27), 10	0.07 (0.2), 20	0.51	0.83	0.60
Mean Δ_30wks-baseline_IL18 (plasma) (SD), *n*	7.59 (89.96), 14	78.68 (161.29), 12	−8.11 (93.54), 14	31.94 (133.95), 26	0.32	0.91	0.64
Mean Δ_30wks-baseline_IL18BP (plasma) (SD), *n*	0.01 (0.24), 14	−0.05 (0.32), 13	0.03 (0.27), 14	−0.01 (0.29), 27	0.83	0.73	0.95
Mean Δ_30wks-baseline_MCP1 (plasma) (SD), *n*	−0.61 (9.41), 14	9.18 (16.23), 13	3.95 (7.85), 14	6.47 (12.63), 27	0.19	0.26	0.15
Mean Δ_30wks-baseline_NFL (CSF) (SD), *n*	1336.44 (2973.3), 7	428.1 (4120.16), 9	−1082.23 (2203.31), 8	−282.64 (3348.96), 17	0.17	0.12	0.09
Mean Δ_30wks-baseline_IL17A (CSF) (SD), *n*	0.11 (0.18), 7	0.02 (0.16), 6	−0.11 (0.22), 7	−0.05 (0.2), 13	0.63	0.05	0.13
Mean Δ_30wks-baseline_IL18 (CSF) (SD), *n*	−0.06 (1.09), 7	−0.3 (1.36), 9	0.56 (1.05), 8	0.1 (1.27), 17	0.54	0.40	0.95
Mean Δ_30wks-baseline_IL18BP (CSF) (SD), *n*	−0.05 (0.47), 7	0.06 (0.41), 9	−0.01 (0.31), 8	0.03 (0.36), 17	0.76	0.87	0.95
Mean Δ_30wks-baseline_MCP1 (CSF) (SD), *n*	13.21 (40.26), 7	28.56 (61.89), 9	−4.19 (34.58), 8	13.15 (52.17), 17	1.00	0.34	0.62
Mean Δ_30wks-baseline_pNFH (CSF) (SD), *n*	−0.15 (0.45), 7	−0.08 (0.27), 9	−0.35 (0.85), 8	−0.21 (0.61), 17	1.00	0.46	0.66
Mean Δ_30wks-baseline_CK (serum) (SD), *n*	43.65 (338.06), 15	−46.5 (408.43), 12	−18.19 (242.79), 14	−31.26 (322.89), 26	0.09	0.20	0.08
Mean Δ_30wks-baseline_creatinine (serum) (SD), *n*	−0.04 (0.21), 15	−0.11 (0.32), 12	−0.08 (0.16), 14	−0.1 (0.24), 26	0.68	0.30	0.38
Mean Δ_30wks-baseline_albumin (serum) (SD), *n*	0.07 (0.39), 15	0 (0.51), 10	0.22 (0.24), 13	0.12 (0.39), 23	0.64	0.21	0.59
Mean Δ_30wks-baseline_vitamin D (serum) (SD), *n*	2.37 (12.91), 15	−4.34 (12.8), 10	0.57 (12.83), 11	−1.77 (12.74), 21	0.09	0.64	0.49

In bold are *P* values statistically significant.

PBMC, peripheral blood mononuclear cell; DRiP, defective ribosomal products; EXO, exosome; MV, microvesicle; NFL, neurofilament light chain; pNFH, phosphorylated neurofilament heavy chain; CK, creatine kinase; SD, standard deviation; Δ_30wks-baseline_, difference in concentrations of each of the biological parameters from baseline to Week 30; ITT, Intention to treat.

^a^The associations between treatment arm and the difference in each of the biological parameters from baseline to Week 30 were assessed using Wilcoxon–Mann–Whitney test. Comparison between colchicine 0.005 mg/kg/day or 0.01 mg/kg/day arms and the placebo arm was corrected using the Bonferroni method to account for multiple arm comparisons; therefore, *P* values are considered statistically significant if <0.025. We did not apply any correction to the comparison of colchicine versus placebo arm; therefore, *P* value is considered statistically significant if <0.05. ITT analysis.

^b^Gene expression data (mRNA) are presented in the form 2^(−ΔΔCt)^, where for each gene and for each patient, the ΔΔCt is calculated as the difference between ΔCt at time 1 (Week 30) and ΔCt at time 0 (baseline). Since the two temporal measures were summarized in a single value, the analysis for repeated measures was not performed. Instead, Mann–Whitney tests were performed to see if the arms of the study had different mean values of 2^(−ΔΔCt)^ for each gene.

We could not detect significant differences across treatment arms in the change from baseline to Weeks 30 and 54 in autophagy pathways as examined by mRNA and protein levels (of HSPA6, SQSTM1/p62, MAP1LC3B, BAG1, BAG3, HSF1, HSPB1 and TFEB) and in peripheral blood mononuclear cells (PBMC) and fibroblasts of amyotrophic lateral sclerosis patients (Supplementary Tables 13 and 14; Supplementary Fig. 5).

Protein levels of SQSTM1/p62, MAP1LC3B (both LC3-I and its lipidated form LC3-II), HSPB8 and BAG3 and levels and relative ratio between soluble and insoluble species of TDP-43, TDP-43 fragments, UBQLN and OPTN were analysed in PBMC in western blot (WB) and filter retardation assay (FRA). Regarding autophagy and co-chaperone proteins, we found a very high degree of variation among patients, without a clear trend of expression level both at Weeks 30 and 54 in PBMC (data not shown). We could not detect HSPB8 protein in all conditions tested. TDP-43 fragmentation analysed in WB was also extremely variable among treated and untreated patients, while the insoluble TDP-43 levels, analysed by FRA, at Week 30 with respect to baseline were increased in the placebo group (MD ± SD: 1.68 ± 2.04) and were decreased in the colchicine group (MD ± SD: −0.21 ± 0.94); this difference was statistically significant (*P* = 0.038). Compared to placebo, there was a decrease in insoluble TDP-43 levels at Week 30 with respect to baseline in the colchicine 0.005 mg/kg/day group (MD −0.63 ± 0.33, *P* = 0.031), but not in the colchicine 0.01 mg/kg/day treated group (MD 0.33 ± 1.25, *P* = 0.177) (Supplementary Table 15). We experienced difficulties in the cell growth procedure of fibroblast cultures prepared to quantify either RNA expression or protein levels. These two assays require a high number of cells. There was high variability among subjects, and we found no differences across treatment arms.

The same variability in defective ribosomal product enrichment inside SGs from fibroblast lines (Supplementary Fig. 6) and the small sample size hampered the identification of significant changes in SG response and composition comparing baseline and Week 30 between placebo and colchicine arms (Supplementary Table 16).

There were no differences among treatment arms in transcriptomic analysis (data not shown), effects on extracellular vesicle secretion (Supplementary Fig. 7; Supplementary Table 17) and WB analysis in exosome contents in TDP-43, TDP-35 and TDP-25 (and their ratio) (Supplementary Fig. 8). No other proteins were detected in exosomes and microvesicles.

Plasma and CSF concentrations of neurodegeneration and inflammation biomarkers (NfL, IL17A, IL18, IL18BP, MCP1 and pNfH) between baseline and Week 30 were not different across treatment arms (Supplementary Tables 18 and 19).

There were not significant differences in peripheral biomarkers (CK, albumin, creatinine and vitamin D) from baseline to Weeks 30 and 54 across treatment arms (Supplementary Table 20).

## Discussion

The phase 2 Co-ALS trial aimed to evaluate the safety, biological and clinical profile of colchicine, a well-known therapeutic agent that may enhance autophagy while simultaneously reducing inflammation, two factors contributing to amyotrophic lateral sclerosis progression. As a non-profit and primarily exploratory study, our focus was on assessing the safety and tolerability of different treatment dosages. The study was designed with high power to detect a 50% difference in the proportion of patients exhibiting an ALSFRS-R decline of fewer than 4 points over 30 weeks between the treatment and placebo arms. Unfortunately, the primary outcome was not met, as only a 20% difference was observed in the low-dose group compared to placebo. The corresponding OR (3.08) had a wide CI, indicating significant uncertainty in the estimated effect.

Despite not meeting the primary objective, patients treated with colchicine at 0.005 mg/kg/day experienced a significantly slower monthly decline in ALSFRS-R during (MD 0.53 points/month) and after treatment (MD 0.46 points/month) compared to placebo. The application of mixed models for repeated measures facilitated the identification of differences even within a smaller cohort^21^; notably, the difference found in terms of the monthly decline in ALSFRS-R is similar to that reported in recent clinical studies.^22,23^ However, no significant impact of colchicine on survival was detected, even in the *post hoc* analysis conducted 1 year following the study’s conclusion. The inconsistency among clinical outcome measures highlights the need for further investigation to elucidate these findings. Factors such as the small sample size (due to an overly optimistic estimation) and the relatively brief treatment duration may have influenced these outcomes.

From a biological point of view, we mainly focused on the drug possible effects on autophagy, finding a trend of decrease in insoluble TDP-43 in PBMC of patients treated with the low dose of colchicine, as evidenced in preclinical studies.^4^ Nevertheless, contrary to our expectations,^3,4,9^ colchicine did not increase the expression of HSPB8 or TFEB in fibroblasts and PBMC. Amyotrophic lateral sclerosis fibroblasts were collected and propagated for several passages without colchicine in the culture medium, both before and during analyses. This implies that any effects of colchicine on fibroblast gene expression may be transient, without the drug priming effects on the selected biochemical markers. Furthermore, the variable effects of colchicine on different human cells and tissues, as well as its varied impacts at different stages of the cell cycle, may explain why no observable effects on fibroblasts were noted.^24^ In addition, mechanisms suggested by preclinical data^9,25^ might not be sufficiently impactful in the *in vivo* context of human disease progression. For example, compensatory mechanisms or other pathogenic amyotrophic lateral sclerosis mechanisms might neutralize the expected benefits of HSPB8 upregulation or autophagy enhancement in patients.^26,27^

Although autophagy pathways and other biological markers did not show notable differences across groups, variations in insoluble TDP-43 levels in PBMC were seen especially in those patients with the slower disease progression (colchicine 0.005 mg/kg/day arm). This result has to be treated with caution due to the limited number of samples analysed, but if confirmed in further studies, understanding the mechanism through which colchicine may reduce TDP-43 accumulation without involving HSPB8 is crucial for therapeutic applications. Since colchicine interferes with microtubule polymerization that is crucial for intracellular transport, colchicine could potentially disrupt the cellular distribution and accumulation of TDP-43. Interestingly a TDP-43 centrosomal enrichment has been recently described.^28^ Colchicine impact on ribosome biogenesis or the localization of ribosomal components (by disrupting microtubules) may potentially influence protein synthesis^11,29^ and might indirectly regulate the production of TDP-43 and therefore its levels in cells, independently of HSPB8.

Finally, colchicine concentrates in immune cells which inhibits the inflammasome and impairs secretion of pro-inflammatory cytokines.^30-32^ By modulating the inflammatory response, colchicine may influence the cellular environment in a way that mitigates TDP-43 pathology.^27,33^ Unfortunately, we could not find a significant change in MCP1, IL-17, IL-18 and IL-18BP in CSF or plasma, and extending the panel of examined neuroinflammation-related biomarkers on a larger cohort would be useful to understand if a potential effect on TDP-43 could be mediated by anti-inflammatory action.

Colchicine treatment was well tolerated, especially at the lower dose. No SAEs were correlated with active treatment. Instead, there was one gastroenteric AE related to the study drug in a patient treated at the 0.01 mg/kg/day dose. Treatment-emergent AEs occurring more frequently in the treated group were gastrointestinal events, detected only in the high-dose group, and due to increased inhibition of mitosis in cells with a high proliferation rate, such as gastrointestinal cells.^34^ We did not observe less common side effects like haematologic or muscular toxicity, but larger studies should be needed to undercover rarer AEs.

Since higher doses of colchicine did not yield any benefits for amyotrophic lateral sclerosis patients, including considerations of safety, the lower dose (0.005 mg/kg/day), which was associated with a reduction in the monthly ALSFRS-R decline and decreased TDP-43 levels, should be considered for further studies.

Colchicine indeed possesses a narrow therapeutic window.^35^ At lower concentrations, it inhibits microtubule aggregation, impacting various cellular processes and pathways to modulate the inflammatory response. At higher concentrations, it promotes microtubule depolymerization, leading to severe toxicity in normal tissues, thereby restricting its broader application.^36^ Consequently, new delivery methods for colchicine are under investigation to mitigate toxicity and enhance its therapeutic efficacy.^35^

In our study, safety was also obtained by excluding patients taking P-glycoprotein (P-gp) and CYP3A4 strong inhibitors, since their concurrent administration could compromise colchicine metabolism, resulting in elevated plasma levels and increased toxicity. The *P-gp/MDR1* gene, known for its high polymorphism, affects the expression and functionality of P-gp and may be linked to colchicine resistance, potentially elucidating the varied response to the drug observed in our study.^36^ MDR1 polymorphism testing should therefore be considered for further studies of colchicine effects in humans.^24,37^

Our study presented some critical issues, such as the small sample size, the short duration and the limited number of samples available at both baseline and end of treatment, partly due to the coronavirus disease 2019 pandemic.

A further critical issue was the drop out number: although we had a number of drop out not larger than previous studies,^38^ it was larger than expected, impacting on outcome measures.

On the other hand, the trial investigated several biomarkers potentially related to amyotrophic lateral sclerosis pathomechanisms, and the results offer valuable insights for further studies.

Future studies should aim to overcome the current study’s limitations, such as small sample size and short follow-up period, and should focus on the lower dose, which showed a better safety profile and some signals of possible benefits for amyotrophic lateral sclerosis patients. Additionally, mechanistic studies are necessary to elucidate the biological pathways influenced by the treatment, which could help in understanding the lack of a dose–response relationship observed. Further research on different pathomechanisms and the findings related to TDP-43 could help clarify colchicine’s mechanism of action in amyotrophic lateral sclerosis.

## Conclusions

In conclusion, colchicine treatment was safe for amyotrophic lateral sclerosis patients. Although some positive signals were observed (such as a reduced rate of decline in the ALSFRS-R score at a specific dosage), the variability across clinical and biological outcomes warrants a cautious interpretation. Additional research is essential to more thoroughly explore colchicine target engagement and its possible impacts on amyotrophic lateral sclerosis patients.

## Supplementary Material

fcae304_Supplementary_Data

## Data Availability

The data that support the findings of this study are available from the corresponding author (jessica.mandrioli@unimore.it) to external researchers who provide methodologically sound scientific proposals and whose proposed use of the data has been approved by an independent review committee identified for this purpose. All requests will be reviewed by the corresponding author and steering committee of the study, and response to requests will be given in 2 months. A material transfer and/or data access agreement with the study promoter will be required for accessing shared data. However, individual participant data will not be available because informed consent did not explicitly include this. The study protocol, including the statistical analysis plan has been uploaded in the Supplementary material file. Source data are provided with this paper.

## Appendix I: Co-ALS investigators group

Jessica Mandrioli, Nicola Fini, Ilaria Martinelli, Elisabetta Zucchi, Giulia Gianferrari, Cecilia Simonini, Francesca Prompicai, Silvia Parisi, Roberto D’Amico, Federico Banchelli, Roberto Vicini, Riccardo Cuoghi Costantini, Angelo Poletti, Valeria Crippa, Elena Casarotto, Serena Carra, Laura Mediani, Francesco Antoniani, Veronica Galli, Valentina Bonetto, Laura Pasetto, Orietta Pansarasa, Eveljn Scarian, Cristina Cereda, Francesca Trojsi, Carla Passaniti, Vincenzo Silani, Nicola Ticozzi, Alberto Doretti, Luca Diamanti, Giuseppe Fiamingo, Mario Sabatelli, Amelia Conte, Giulia Bisogni, Giuseppe Lauria, Eleonora Dalla Bella, Nilo Riva, Enrica Bersano, Isabella Laura Simone and Eustachio D’Errico
