# Supplementary material for: Colchicine treatment in amyotrophic lateral sclerosis: safety, biological and clinical effects in a randomized clinical trial
Source: Brain Commun. 2024 Sep 5;6(5):fcae304. doi: 10.1093/braincomms/fcae304 (PMC11406549; doi:10.1093/braincomms/fcae304)
Supplement: fcae304_Supplementary_Data [file fcae304_supplementary_data.zip › Supplementary_material.pdf]

SUPPLEMENTARY MATERIAL

This additional file has been provided by the authors to give readers additional information about their work.

Supplement to:

Colchicine treatment in Amyotrophic Lateral Sclerosis: safety, biological and clinical effects in a randomized clinical trial.

Giulia Gianferrari[1,2*], MD, Riccardo Cuoghi Costantini[3*], MSc, Valeria Crippa[4*], PhD, Serena Carra[1], PhD, Valentina Bonetto[5], PhD, Orietta Pansarasa[6], PhD, Cristina Cereda[7], MD, Elisabetta Zucchi[2,8], MD, Ilaria Martinelli[2,9], MD, Cecilia Simonini[2],BSc, Roberto Vicini[3], PhD, Nicola Fini, MD[2], Francesca Trojsi[10], PhD, Carla Passaniti[10], MD, Nicola Ticozzi[11,12], MD, Alberto Doretti[11], MD, Luca Diamanti[13], MD, Giuseppe Fiamingo[13], MD, Amelia Conte[14], PhD, Eleonora Dalla Bella[15], MD, Eustachio D'Errico[16], MD, Eveljn Scarian[6], PhD, Laura Pasetto[5], PhD, Francesco Antoniani[1], PhD, Veronica Galli[1], PhD, Elena Casarotto[4], PhD, Co-ALS investigators group, Prof Roberto D'Amico[3,17#], PhD, Prof Angelo Poletti[4#], PhD, Jessica Mandrioli[1,2#], MD

[1] Department of Biomedical, Metabolic and Neural Sciences, University of Modena and Reggio Emilia, Modena, 41121, Italy

[2]Department of Neurosciences, Azienda Ospedaliero Universitaria di Modena, Modena, 41126, Italy

[3]Unit of Statistical and Methodological Support to Clinical Research, Azienda Ospedaliero-Universitaria, Modena, 41121, Italy.

[4]Dipartimento di Scienze Farmacologiche e Biomolecolari "Rodolfo Paoletti", Dipartimento di Eccellenza 2018-2027, Università degli Studi di Milano, Milan, 20122, Italy.

[5] Research Center fo ALS, Istituto di Ricerche Farmacologiche Mario Negri IRCCS, Milan, 20156, Italy

[6]Cellular Model and Neuroepigenetics Unit, IRCCS Mondino Foundation, Pavia, 27100, Italy.

[7]Center of Functional Genomics and Rare diseases, Department of Pediatrics, "V. Buzzi" Children's Hospital, Milan, 20154, Italy.

[8]Neurosciences PhD Program, University of Modena and Reggio Emilia, Modena, 41121, Italy

[9]Clinical and Experimental Medicine PhD Program, University of Modena and Reggio Emilia, Modena, 41121, Italy

[10] ALS Center, Department of Advanced Medical and Surgical Sciences, Università degli Studi della Campania L. Vanvitelli, Naples, 80138, Italy.

[11] Department of Neurology, IRCCS Istituto Auxologico Italiano, Milan, 20149, Italy

[12] Department of Pathophysiology and Transplantation, 'Dino Ferrari' Center, Università degli Studi di Milano, Milan, 20122, Italy

[13]IRCCS Mondino Foundation, Pavia, 27100, Italy.

[14]Adult NEMO Clinical Center, Unit of Neurology, Department of Aging, Neurological, Orthopedic and Head-Neck Sciences, Fondazione Policlinico Universitario A. Gemelli IRCCS, Rome, 00168, Italy.

[15]3rd Neurology Unit and Motor Neuron Disease Centre, Fondazione IRCCS Istituto Neurologico Carlo Besta, Milan, 20133, Italy.

[16]ALS Center, Department of Basic Medical Sciences, Neurosciences and Sense Organs, University of Bari, Bari, 70124, Italy.

[17]Department of Medical and Surgical Sciences for Children and Adults, University of Modena and Reggio Emilia, Modena, 41124, Italy.

[*] Giulia Gianferrari, Riccardo Cuoghi Costantini and Valeria Crippa contributed equally to this work

[#] Roberto D'Amico, Angelo Poletti and Jessica Mandrioli contributed equally to this work

Corresponding author:

Jessica Mandrioli

Department of Biomedical, Metabolic and Neural Sciences, University of Modena and Reggio Emilia, Modena, Italy;

Via Pietro Giardini n. 1355

41126 Modena, Italy

Tel. +390593961640 Fax +390593963775

Cell +393392633246

E-Mail jessica.mandrioli@unimore.it

Table of contents

1. Clinical Trial Sites and Site Investigators 6

2. Supplementary Methods 8

Biological outcome measures: laboratory methods 8

Section 2.1 Samples preparation and storage 8

Section 2.2 mRNA and protein levels of p62, LC3, TFEB, ATGs, HSPB8, BAG3, BAG1, HSPA6, and HSF1 on fibroblasts and mononuclear blood cells as indicators of autophagy and cell response to stress 9

Section 2.3 Labeling of Nascent Peptides with op-PURO and analysis of stress granules response and composition on fibroblasts and mononuclear blood cells 10

Section 2.4 Levels and relative ratio between soluble and insoluble species of TDP-43, TDP-43 fragments, SQSTM1/p62, UBQLN, OPTN on fibroblasts and mononuclear blood cells . 10

Section 2.5 Extracellular vesicles isolation from plasma and CSF with analysis of their content of hyperphosphorylated TDP-43, SQSTM1/p62, UBQLN and OPTN 11

Section 2.6 RNA-SEQ analysis and validation 11

Section 2.7 Neurofilament and other biomarkers quantification 12

3. Supplementary Tables 13

Supplementary Table 1. Biological features of patients enrolled in the three trial arms at baseline (plasma) 13

Supplementary Table 2. Biological features of patients enrolled in the three trial arms at baseline (CSF) 14

Supplementary Table 3. Biological features of patients enrolled in the three trial arms at baseline (fibroblasts)……………………………………………………………………...…..15

Supplementary Table 4: Individuals with Adverse Events (AEs) across different treatment arms 16

Supplementary Table 5: Individuals with Serious Adverse Events (SAEs) across different treatment arms 17

Supplementary Table 6: Adverse Events across different treatment arms.................................... 18

Supplementary Table 7: Deaths occurring during the study 19

Supplementary Table 8. Absolute changes from baseline to week 4-12-18-24-30-36-42-54 in ALSFRS-R total score in patients treated with colchicine or placebo. 20

Supplementary Table 9. Logrank test and Cox proportional hazard model for tracheostomy-free survival in patients enrolled in co-ALS trial across treatment arms. 22

Supplementary Table 10. Changes in the respiratory function as measured by forced vital capacity

(FVC%) from baseline to different time points in the three treatment arms. 23
Supplementary Table 11. ALSAQ40 administered at different time points in patients treated with colchicine and placebo. 25
Supplementary Table 12: Changes in clinical outcome measures during and after treatment across treatment arms 29
Supplementary Table 13: Changes in mRNA contents in PBMC comparing baseline and week 30.…... …32
Supplementary Table 14: Changes in mRNA contents in PMBC comparing baseline and week 54.…… 34
Supplementary Table 15: Changes in insoluble TDP43 protein levels in PBMC at baseline (T0) and week 30 (T1) in placebo and colchicine-treated groups. 36
Supplementary Table 16: Quantity of DRiPs inside SGs at baseline (T0) and week 30 (T1) across treatment arms. 37
Supplementary Table 17: Exosomes (EXOs) and microvescicles (MVs) plasma concentration across treatment arms at baseline (T0) and week 30 (T1). 39
Supplementary Table 18. Changes of biomarkers of neuroinflammation and neurodegeneration across treatment arms between baseline (T0) and week 30 (T1) and week 54 (T2) in plasma 40
Supplementary Table 19. Changes of biomarkers of neuroinflammation and neurodegeneration across treatment arms between baseline (T0) and week 30 (T1) in CSF 44
Supplementary Table 20: Mean difference from baseline to week 30 and 54 in CK, albumin, creatinine and vitamin D between colchicine and placebo arms 47
4. Supplementary Figures 50
Supplementary Figure 1. Individual rates of decline in ALSFRS-R total score of patients enrolled in co-ALS over the study 50
Supplementary Figure 2. Tracheostomy-free survival from baseline based on treatment arm allocation. 51
Supplementary Figure 3. Respiratory muscle function as assessed by forced vital capacity score decline from baseline to weeks 4, 12, 18, 24, 30, 42, and 54 across treatment arms…………………………………………………………………………………….52
Supplementary Figure 4. Mean scores of ALSAQ40 from baseline to the end of the study across treatment arms 53
Supplementary Figure 5. Effects on autophagy related gene (HSPB1, BAG3, BAG1, HSF1, TFEB, SQSTM1 p62, MAP1LC3B, HSPA6) expression by different doses of colchicine

in patients PBMCs at week 30 with respect to baseline ... 54
Supplementary Figure 6. Enrichment of DRiPs inside arsenite-induced SGs in fibroblasts from ALS patients at baseline and treatment end. ... 55
Supplementary Figure 7. Exosomes (EXOs) and microvescicles (MVs) production from patients' plasma across treatment arms at baseline (T0) and week 30 (T1). ... 56
Supplementary Figure 8. Western Blot analysis in exosomes (EXOs) from patients' plasma measuring TDP-43, TDP-35, and TDP- 25 expressions across treatment arms at baseline (T0), week 30 (T1) and week 54 (T2). ... 57
4. References ... 59
5. Abbreviations List ... 60

1. Clinical Trial Sites and Site Investigators

Trial Sites and Locations	Principal Investigator	Sub-investigators and collaborators
**Modena**		
ALS Center, Azienda Ospedaliero Universitaria di Modena, Modena, Italy	Jessica Mandrioli, M.D.	Nicola Fini,<br>M.D. Ilaria<br>Martinelli,<br>M.D.<br>Elisabetta Zucchi,<br>M.D. Giulia<br>Gianferrari, M.D.<br>Cecilia Simonini,<br>BSc Roberta<br>Bedin, BSc<br>Francesca<br>Prompicai, R.N.<br>Silvia Parisi, R.N.
**Naples**		
ALS Center, Department of Advanced Medical and Surgical Sciences,<br>l, Università degli Studi della Campania L. Vanvitelli, Naples, Italy.	Francesca Trojsi, PhD.	Carla Passaniti, M.D.
**Bari**		
ALS Centre, University of Bari, Bari, Italy	Isabella Laura Simone, M.D.	Eustachio D’Errico, Ph.D.
**Pavia**		
ALS Center, IRCCS Istituto Neurologico Nazionale “C. Mondino”<br>Pavia, Italy	Luca Diamanti, M.D.	Giuseppe Flamingo, M.D.

**Rome**		
Adult NEMO Clinical Center, Unit of Neurology, Department of Aging, Neurological, Orthopedic and Head-Neck Sciences, Fondazione Policlinico Universitario A. Gemelli IRCCS, Rome, Italy.	Mario Sabatelli, Ph.D.	Amelia Conte, M.D., Giulia Bisogni, M.D.
**Milan**		
ALS Center, Istituto Auxologico di Milano, University of Milan, Italy.	Vincenzo Silani, M.D.	Nicola Ticozzi, M.D. Alberto Doretti, M.D.
3rd Neurology Unit and ALS Centre, IRCCS Istituto Neurologico Carlo Besta di Milano, Milan, Italy	Eleonora Dalla Bella, M.D.	Giuseppe Lauria Pinter, M.D. Enrica Bersano, M.D.
IRCCS Istituto Neurologico San Raffaele di Milano, Milan, Italy	Nilo Riva, MD	

2. Supplementary Methods

Biological outcome measures: laboratory methods

Section 2.1 Samples preparation and storage

Forty milliliters of blood were collected in vacuette containing EDTA and immediately processed according to biosafety rules to obtain the following samples.

Serum isolation was obtained from venous blood collected in 1x 10-ml silica vacutainer; samples were allowed to clot for 1- 2 hours on ice at 4° C. Tubes were centrifuged at 1500 x g for 15 min at room temperature and stored at -80° C until analysis. Plasma isolation was obtained from blood collected in 4 x 5-ml EDTA vacutainers and centrifugated at 2500 x g for 15 min at room temperature. Plasma was aliquoted into cryo tubes and stored at -80° C until analysis (storage in single clinical center then shipment to the Laboratory of Translational Biomarkers, Istituto di Ricerche Farmacologiche Mario Negri IRCCS, Milano).

Other aliquots were obtained from venous blood collected in sodium citrate vacutainers (2x 5 ml Na) and centrifugated at 1000 x g for 15 min within 1 h, followed by an additional centrifugation at 1600 x g for 20 min. Platelet-free plasma was frozen at -80°C until analysis (storage in single clinical center then shipment to IRCCS “C. Mondino” National Neurological Institute Foundation, Pavia)

Peripheral blood mononuclear cells (PBMCs) isolation was obtained from blood collected in 3x 9ml EDTA vacutainer. Isolation of PBMCs was performed by using Ficoll-Histopaque®-1077 (Sigma-Aldrich, Milan, Italy) according to standard procedures[1]. PBMCs were used as freshly isolated for mononuclear blood cells or stored in liquid nitrogen in FBS added with 10% DMSO for B cell analysis. Plasma was collected, centrifuged at 2500 x g, and stored at−80°C until use.

Cerebrospinal fluid (CSF) was collected in polypropylene tubes, centrifuged at 450 x g for 10 min at room temperature and stored at -80° C until analysis (storage in single clinical center then shipment to Laboratory of Translational Biomarkers, Istituto di Ricerche Farmacologiche Mario Negri IRCCS, Milano and to Cellular Model and Neuroepigenetics Unit “C. Mondino” National Neurological Institute, Pavia).

Fibroblasts were generated from skin biopsies; the tissue was cut into small fragments and distributed over the bottom surface of the culture flask. Fibroblasts started to grow in 6 days and then cells were plated for proliferation. Aliquots of fibroblasts were stored at -80 ° C until analysis. Aliquots were sent to the other Units to perform scientific experiments. Cells used in the experiments were grown and

expanded to a maximum of thirteen passages.

Section 2.2 mRNA and protein levels of p62, LC3, TFEB, ATGs, HSPB8, BAG3, BAG1, HSPA6, and HSF1 on fibroblasts and mononuclear blood cells as indicators of autophagy and cell response to stress

To evaluate the colchicine efficacy in activating the autophagy pathway and/or a response to stress, mRNA and protein levels were isolated from patients' cells and investigated by RT-qPCR and Western Blot (WB) analysis. Total RNA was isolated from PBMCs and fibroblasts using Tri-Reagent (Merck Millipore, Rahway, NJ, USA) and following manufacturer's specifications. A NanoDrop 2000 (ThermoFisher Scientific, Waltham, MA, USA) was used for RNA quantification and 1 µg per sample was treated with DNAse and reverse transcribed using the High-Capacity cDNA Reverse Transcription Kit (ThermoFisher Scientific, Waltham, MA, USA) according to the manufacturer's instructions. The following primer were used: hHSPB8, hBAG1, hBAG3, hHSF1, hTFEB, hMAP1LC3B and hSQSTM1/p62[1], hHSPA6 (forward 5'- TGA ATG GCC CTT CGT GAT AAG-3', reverse 5'-TTG GAG GGA AAG TTC ATC TCT GA-3') and hHSPB1 (forward 5'- ACG CAGTCC AAC GAG ATC AC-3', reverse 5'-TTT ACT TGG CGC CAG TCT CAT-3'). RT-qPCR was performed using the CFX 96 Real-Time System (Bio-Rad Laboratories, Hercules, CA, USA) in a 10 µL total volume, with 500 nmol primers, using the iTaq SYBR Green Supermix (Bio-Rad Laboratories, Hercules, CA, USA), as previously described[2]. Data were normalized to the amount of the housekeeping gene ribosomal protein lateral stalk subunit P0 (Rplp0). Sample Ct values were used for target relative quantification through the ΔΔCt calculation. The N-fold changes in gene expression were obtained transforming data by the equation 2−ΔΔCt.

For protein analyses, cell pellets were lysed in RIPA buffer (0.15 M NaCl, 0.8% sodium deoxycholate, 100 µM sodium orthovanadate, 50 mM NaF, 5 mM sodium iodoacetate, 0.05 M Tris HCl (pH 7.7), 10 mM EDTA (pH 8), 0.08% SDS and Triton X-100 supplemented with protease inhibitors (complete tablets, Roche Diagnostics GmbH, Mannheim, Germany) for 20 min on ice and then slightly sonicated. RIPA extracts were quantified with the bicinchoninic acid assay (BCA) (Cyanagen Reagents for Molecular Biology, Bologna, Italy) and 15 µg of proteins was loaded on 12% SDS-polyacrylamide gel, resolved by electrophoresis, and transferred to 0.45 µm nitrocellulose membrane (ThermoFisher Scientific, Waltham, MA, USA) with the Trans-Blot® Turbo™ transfer system (Bio-Rad Laboratories, Hercules, CA, USA).

Nitrocellulose membranes were processed as in[3] with the following primary and secondary antibodies:

GAPDH (MAB-10578, dilution 1:3000; Immunological Science, Roma, Italy), BAG3 (ab47124, dilution 1:1000; Abcam, Cambridge, UK), HSPB8 (PA5-76780, dilution 1:1000, Thermo Fisher), MAP1LC3B (L8918, dilution 1:1000, Merck Millipore, Rahway, NJ, USA), SQTM1/p62 (P0067, dilution 1:1000, Merck Millipore, Rahway, NJ, USA) and Goat Anti-Rabbit-HRP conjugated antibody (111-035-003, dilution 1:5000, Jackson Immuno Research Laboratories, PA, USA). Chemiluminescent signals were detected using the enhanced chemiluminescence detection kit reagents Westar Antares (Cyanagen, Bologna, Italy). A ChemiDoc XRS+ System (Bio-Rad Laboratories, Hercules, CA, USA) was used for image acquisition. Optical intensity of the assayed samples was detected and analyzed using the Image Lab software version 5.2.1 (Bio-Rad Laboratories, Hercules, CA, USA).

Section 2.3 Labeling of Nascent Peptides with op-PURO and analysis of stress granules response and composition on fibroblasts and mononuclear blood cells

The protocol for op-PURO labeling was previously described[4]. Fibroblast lines were treated with sodium arsenite (0.5mM Carlo Erba Reagents, Cornaredo, Milano, Italy) and OP-puro (25 μM) for 45 min, immediately fixed or let to recover in growth medium.

High content imaging-based assay

Images were obtained using a Leica SP8 confocal microscope equipped with a 405 nm and white light lasers using a 63× oil immersion objective. Stress granules (SGs) composition was analysed using the Scan^R Analysis software (Olympus). First, SGs were segmented based on G3BP1 signal using edge detection algorithm. The mean fluorescence intensity of the protein of interest (DRiPs) was then measured in each detected SGs. The mean fluorescent intensity of DRiPs was also measured in an area surrounding the SG. The ratio of mean fluorescence intensity inside the SGs divided by mean intensity in the region surrounding the SGs represents the relative enrichment of DRiPs in individual SGs. The values were plotted as histograms or as column graphs representing the fraction of SGs with enrichment > 1.5. Statistical analyses were performed using one-way ANOVA, followed by Bonferroni–Holm post hoc test for comparisons between three or more groups.

Section 2.4 Levels and relative ratio between soluble and insoluble species of TDP-43, TDP-43 fragments, SQSTM1/p62, UBQLN, OPTN on fibroblasts and mononuclear blood cells

TDP-43 ALS-related fragmentation was investigated in WB taking advantage of the TDP C-Terminal antibody (12892-1-AP, dilution 1:1000, Proteintech, Manchester, UK), that specifically recognize the C-terminal region of the protein, while TDP- 43 insoluble species accumulation was tested in filter

retardation assay (FRA). For WB analysis, cell proteins were extracted in RIPA buffer and processed as described in Section 2.3.2. For FRA, 3 μg of RIPA protein extract were diluted in 100 μl of RIPA buffer, loaded in a dot-blot apparatus (Bio-Rad Laboratories, Hercules, CA, USA) and filtered through a 0.22 μm cellulose acetate membrane (Whatman, Maidstone, UK). Proteins were then fixed using 99% methanol and processed as for WB.

TDP-43 fragmentation resulted extremely variable among patients (both treated and untreated samples). Similarly, the levels of the main autophagy receptor SQSTM1/p62 considerably varied among patients. Optineurin and ubiliquin 2 were not tested because of this high variability among subjects in SQSTM1/p62.

Section 2.5 Extracellular vesicles isolation from plasma and CSF with analysis of their content of hyperphosphorylated TDP-43, SQSTM1/p62, UBQLN and OPTN

Exosomes (EXOs) from plasma (at least 3 ml) were isolated by sequential centrifugations to remove first floating cells and debris. Medium will be stored at −80 °C until extracellular vesicles isolation. After microvesicles (MVs) isolation, the resulting supernatant was ultracentrifuged to pellet exosomes, as previously described[5]. Exosomes were characterized by immunoblot analysis of exosomal biomarkers such as Alix, and by a Nanoparticle Tracking Analysis instrument (LM10HSBF, NanoSight ltd, Malvern, United Kingdom). Vesicles were further washed, re-suspended in cold RIPA buffer (+PIC+PhosphoStop) and stored at −80 °C. Protein concentration were measured by BCA assay and immunoblot analysis for TDP-43, TDP-43 fragments, HSC70, HSPB8 as well as exosomes markers (Alix) and microvesicles markers (Annexin V). Extracellular vesicles secretion in CSF could not be evaluated because of insufficient amount of CSF due to low concentration of EXOs in CSF[6].

Section 2.6 RNA-SEQ analysis and validation

Total RNA was extracted from PBMCs using TRIzol® (Invitrogen) following the manufacturer's instructions, analyzed by a Bioanalyzer spectrophotometer, and stored at -80°C until use. RNA quantification was carried out by absorbance at 260 nm. RNA sequencing libraries were prepared using the Illumina TruSeq Stranded RNA Library Prep (Illumina). RNA processing were carried out using Illumina NextSeq 500 Sequencing. The output trascriptome file results (Target Hits Per Sample) were used for differential expression analysis (Edger package, BioConductor, R).Total RNA (1 mg) was reverse transcribed into cDNA using the iScriptcDNA Synthesis Kit (BioRad, according to the manufacturer's protocol. Validation primers for autophagy pathway were: hHSPB8, hBAG1, hBAG3,

hp62/SQSTM1, hMAP-LC3-B, hTFEB, hBECLIN1, hRplPO.

Data were expressed as threshold cycle (Ct) values and used for the relative quantification of targets with the ΔΔCt calculation. Next, data were transformed through the equation $2^{-\Delta\Delta Ct}$ to give N-fold changes in gene expression, all statistics was performed with ΔCt values.

Section 2.7 Neurofilament and other biomarkers quantification

CSF and plasma samples were collected, processed, and stored at -80° C, until further analysis. MCP-1 and IL-18BP concentration were measured in both biofluids using AlphaLISA kits (Perkin Elmer, Waltham, MA, USA) No. AL244C and No. AL3096C respectively, assisted by the Zephyr® liquid handler (Perkin Elmer, Waltham, MA, USA). The AlphaLISA signals were detected by an Ensight Multimode Plate Reader (Perkin Elmer, Waltham, MA, USA). NfL and IL-17A concentrations were measured in CSF and plasma using the Simoa® kits No. 103400 and No. 101599 respectively, on the Quanterix SR-XTM platform according to the protocol issued by the manufacturer (Quanterix Corp, Boston, MA, USA). IL-18 was measured in CSF by a Simoa® kits No. 102700 on the Quanterix SR-XTM platform, in plasma by an AlphaLISA kit (Perkin Elmer, Waltham, MA, USA) No. AL3137C. pNFH was measured in CSF with an ELISA kit for the human protein (EUROIMMUN #EQ-6561-9601). In each analysis sample were measured in duplicate.

3. Supplementary Tables

Supplementary Table 1. Biological features of patients enrolled in the three trial arms at baseline (plasma)

		**Placebo (n = 18)**			**Colchicine 0.005 mg/kg/d (n = 18)**			**Colchicine 0.01 mg/kg/d (n = 18)**		
		N	mean	SD	N	mean	SD	N	mean	SD
CK	UI/ml	18	235.91	291.79	18	336.39	285.67	18	320.22	205.96
Albumin	mg/dl	18	4.26	0.30	18	4.31	0.31	18	4.18	0.22
Creatinine	mg/dl	18	0.72	0.17	18	0.69	0.19	18	0.74	0.14
Vit D	ng/dl	18	27.89	8.68	18	27.43	10.88	18	26.35	9.19
IL-17A, plasma	pg/ml	13*	0.20	0.22	15*	0.14	0.13	13*	0.20	0.26
IL-18, plasma	pg/ml	18	382.68	177.07	18	277.83	114.59	18	375.88	168.82
IL-18BP, plasma	ng/ml	18	1.71	0.32	18	1.76	0.28	18	1.61	0.27
MCP1, plasma	pg/ml	18	37.06	15.46	18	34.66	12.93	18	38.23	14.78
NfL, plasma	pg/ml	18	138.37	141.72	18	79.07	33.64	18	109.58	71.28
Exosomes, plasma	EXO/ml	7	-0.17	1.12	8	-0.03	0.96	8	0.14	1.42
Microvescicles, plasma	MV/ml	7	-0.02	1.35	8	-0.35	0.62	8	-0.22	0.85

* 54 samples collected: the missing values for IL-17A are related to samples with concentrations below the detection limit. UI = international unit. EXO =exososome. MV = microvescicle. SD = standard deviation.

Supplementary Table 2. Biological features of patients enrolled in the three trial arms at baseline (CSF)

		Placebo (n = 18)			Colchicine 0.005 mg/kg/d (n = 18)			Colchicine 0.01 mg/kg/d (n = 18)		
		N	mean	SD	N	mean	SD	N	mean	SD
IL-17A, CSF	pg/ml	12*	0.42	0.19	10*	0.36	0.25	16*	0.42	0.16
IL-18, CSF	pg/ml	16	3.47	2.17	15	3.33	1.39	17	3.33	1.38
IL-18BP, CSF	ng/ml	16	4.40	1.13	15	4.38	1.26	17	4.19	0.82
MCP1, CSF	pg/ml	16	261.61	68.69	15	295.35	114.95	17	300.66	76.81
NfL CSF	pg/ml	16	8283.91	6568.07	15	7170.23	2828.21	17	8199.19	5498.24
pNfH CSF	ng/ml	16	3.29	2.53	15	2.26	1.15	17	2.65	1.48

*48 samples collected: the missing values for IL-17A are related to samples with concentrations below the detection limit. SD = standard deviation.

Supplementary Table 3. Biological features of patients enrolled in the three trial arms at baseline (fibroblasts)

		Placebo (n = 18)		Colchicine 0.005 mg/kg/d (n = 18)		Colchicine 0.01 mg/kg/d (n = 18)	
		mean	SD	mean	SD	mean	SD
DRiPs	AU	1.27	0.15	1.28	0.12	1.27	0.23
DRiPs 0-1,5 (%)	AU	85.60	8.04	85.55	5.77	84.93	11.42
DRiPs 1,5-3,0 (%)	AU	13.57	7.44	13.04	4.79	13.77	10.02
DRiPs <3,0 (%)	AU	0.79	0.79	1.24	1.02	1.28	1.44

DRiPs = Defective Ribosomal Products; AU= Arbitrary Unit (see methods section). SD = standard deviation.

Supplementary Table 4: Individuals with Adverse Events (AEs) across different treatment arms

The unadjusted comparisons were carried out with a chi-square test without any correction. Comparison between colchicine 0.005 mg/Kg/d or 0.01 mg/Kg/d arms and the placebo arm was carried out using the Bonferroni method to account for multiple arm comparisons, therefore Confidence Intervals (CI) are set at 97.5% and p value are considered statistically significant if <0.025. We did not apply any correction to the comparison of colchicine versus placebo arm, therefore CI are set at 95% and p value are considered statistically significant if <0.05.

Time point	Treatment arm	AEs n	AEs %	No AEs n	No AEs %	RR	CI	p value
Week 30	Placebo	8	44.4%	10	55.6%	-	-	-
	Colchicine 0.005 mg/kg/d	3	16.7%	15	83.3%	0.38	0.11 - 1.19	0.070
	Colchicine 0.01 mg/kg/d	7	38.9%	11	61.1%	0.88	0.40 - 1.90	0.735
	Colchicine	10	27.8%	26	72.2%	0.63	0.30 - 1.31	0.221
Week 54	Placebo	9	50.0%	9	50.0%	-	-	-
	Colchicine 0.005 mg/kg/d	7	38.9%	11	61.1%	0.78	0.37 - 1.63	0.502
	Colchicine 0.01 mg/kg/d	13	72.1%	5	27.8%	1.44	0.84 - 2.49	0.171
	Colchicine	20	55.6%	16	44.4%	1.11	0.64 - 1.91	0.700

AEs = adverse events; RR = relative risk; CI = confidence interval.

Supplementary Table 5: Individuals with Serious Adverse Events (SAEs) across different treatment arms

The unadjusted comparisons were carried out with a chi-square test without any correction. Comparison between colchicine 0.005 mg/Kg/d or 0.01 mg/Kg/d arms and the placebo arm was carried out using the Bonferroni method to account for multiple arm comparisons, therefore Confidence Intervals (CI) are set at 97.5% and p value are considered statistically significant if <0.025. We did not apply any correction to the comparison of colchicine versus placebo arm, therefore CI are set at 95% and p value are considered statistically significant if <0.05.

Time point	Treatment arm	SAEs		No SAEs		RR	CI	p value
		n	%	n	%			
Week 30	Placebo	3	16.7%	15	83.3%	-	-	-
	Colchicine 0.005 mg/kg/d	1	5.6%	17	94.4%	0.33	0.04 - 2.91	0.289
	Colchicine 0.01 mg/kg/d	3	16.7%	15	83.3%	1.00	0.23 - 4.30	1.000
	Colchicine	4	11.1%	32	88.9%	0.67	0.17 - 2.67	0.567
Week 54	Placebo	6	33.3%	12	66.7%	-	-	-
	Colchicine 0.005 mg/kg/d	5	27.8%	13	72.1%	0.83	0.31 - 2.24	0.718
	Colchicine 0.01 mg/kg/d	7	38.9%	11	61.1%	1.16	0.49 - 2.79	0.729
	Colchicine	12	33.3%	24	66.7%	1.00	0.45 - 2.23	1.000

SAEs = serious adverse events; RR = relative risk; CI = confidence interval.

Supplementary Table 6: Adverse Events across different treatment arms

		Placebo (n = 18)		Colchicine 0.005 mg/kg/d (n = 18)		Colchicine 0.01 mg/kg/d (n = 18)	
AE	n	14		15		19	
SAE	n % [a]	6	42.8%	5	33.3%	7	36.8%
Relationship with treatment (none/remote)	n % [a]	14	100.0%	14	93.3%	18	94.7%
AE leading to treatment discontinuation	n % [a]	0	0.0%	1	6.6%	1	5.3%

AE = adverse event; SAE = serious adverse event; [a] = % of AEs

Supplementary Table 7: Deaths occurring during the study

Time point	Treatment arm	Deaths n	Deaths %	Cause of death	Relationship to study drug
Baseline to Week 30	Placebo	1	5.5%	Respiratory failure	None
	Colchicine 0.005 mg/kg/d	1	5.5%	Suicide	None
	Colchicine 0.01 mg/kg/d	1	5.5%	Respiratory failure	None
Week 30 to 54	Placebo	2	11.1%	Respiratory failure in both cases	None
	Colchicine 0.005 mg/kg/d	2	11.1%	Cardiac arrest in both cases	None
	Colchicine 0.01 mg/kg/d	1	5.5%	Respiratory failure	None

Supplementary Table 8. Absolute changes from baseline to week 4-12-18-24-30-36-42-54 in ALSFRS-R total score in patients treated with colchicine or placebo.

Mean absolute changes from baseline to week 12-18-24-30-36-42-54 are showed for each treatment group; comparison were performed using linear regression models that include indicator variables for treatment arms as the independent variables. Comparison between colchicine 0.005 mg/Kg/d or 0.01 mg/Kg/d arms and the placebo arm was carried out using the Bonferroni method to account for multiple arm comparisons, therefore Confidence Intervals (CI) are set at 97.5% and p value are considered statistically significant if <0.025. We did not apply any correction to the comparison of colchicine versus placebo arm, therefore CI are set at 95% and p value are considered statistically significant if <0.05.

**Time point**	**Arm**	**Absolute change from baseline**						
		**n**	**mean**	**SD**	**MD**	**CI**		**p value**
Week 4	Placebo	18	1.722	1.776				
	Colchicine 0.005 mg/kg/d	18	0.778	1.517	-0.944	-2.351	0.462	0.138
	Colchicine 0.01 mg/kg/d	18	1.667	2.275	-0.056	-1.462	1.351	0.930
	Colchicine	36	1.222	1.958	-0.500	-1.575	0.575	0.366
Week 12	Placebo	18	6.667	5.314				
	Colchicine 0.005 mg/kg/d	16	3.688	2.626	-2.979	-6.353	0.394	0.054
	Colchicine 0.01 mg/kg/d	17	4.706	4.593	-1.961	-5.281	1.36	0.192
	Colchicine	33	4.212	3.748	-2.455	-4.956	0.047	0.060
Week 18	Placebo	17	7.647	6.461				
	Colchicine 0.005 mg/kg/d	17	5.176	3.941	-2.471	-6.651	1.71	0.191
	Colchicine 0.01 mg/kg/d	18	7.500	5.597	-0.147	-4.269	3.975	0.937

	Colchicine	35	6.371	4.935	-1.276	-4.445	1.894	0.434
Week 24	Placebo	16	9.750	7.971				
	Colchicine 0.005 mg/kg/d	16	5.563	4.226	-4.188	-9.074	0.699	0.061
	Colchicine 0.01 mg/kg/d	17	9.176	5.747	-0.574	-5.388	4.241	0.791
	Colchicine	33	7.424	5.315	-2.326	-6.079	1.427	0.231
Week 30	Placebo	15	9.667	6.433				
	Colchicine 0.005 mg/kg/d	15	7.133	4.704	-2.533	-6.961	1.894	0.207
	Colchicine 0.01 mg/kg/d	14	8.929	4.891	-0.738	-5.244	3.768	0.715
	Colchicine	29	8.000	4.796	-1.667	-5.031	1.698	0.337
Week 36	Placebo	14	10.857	6.982				
	Colchicine 0.005 mg/kg/d	13	8.000	5.244	-2.857	-8.15	2.435	0.234
	Colchicine 0.01 mg/kg/d	14	11.214	5.977	0.357	-4.836	5.551	0.878
	Colchicine	27	9.667	5.765	-1.190	-5.191	2.810	0.563
Week 42	Placebo	13	11.154	6.517				
	Colchicine 0.005 mg/kg/d	11	7.455	5.126	-3.699	-9.534	2.135	0.165
	Colchicine 0.01 mg/kg/d	12	11.000	7.135	-0.154	-5.855	5.548	0.952
	Colchicine	23	9.304	6.378	-1.849	-6.221	2.522	0.413
Week 54	Placebo	11	11.000	4.427				
	Colchicine 0.005 mg/kg/d	11	9.818	5.671	-1.182	-6.238	3.874	0.604
	Colchicine 0.01 mg/kg/d	9	11.333	5.766	0.333	-4.996	5.663	0.890
	Colchicine	20	10.500	5.615	-0.500	-4.352	3.352	0.801

SD = standard deviation; MD = mean difference; CI = confidence interval.

Supplementary Table 9. Logrank test and Cox proportional hazard model for tracheostomy-free survival in patients enrolled in co-ALS trial across treatment arms.

Arm	Baseline subjects	Observed events (Deaths or Tracheostomies)	Expected events	Log-Rank test p-value
Placebo	18	6 (3-3)	4.49	-
Colchicine 0.005 mg/kg/d	18	3 (3-0)	4.71	0.550
Colchicine 0.01 mg/kg/d	18	4 (2-2)	3.80	
Colchicine	36	7 (5-2)	8.51	0.389
	**HR**	**95% CI**		**p-value**
Placebo	Reference			
Colchicine 0.005 mg/kg/d	0.477	0.119	1.908	0.295
Colchicine 0.01 mg/kg/d	0.791	0.222	2.812	0.717
Colchicine	0.616	0.207	1.835	0.384

HR = Hazard Ratio; CI = confidence interval

Supplementary Table 10. Changes in the respiratory function as measured by forced vital capacity (FVC%) from baseline to different time points in the three treatment arms.

Comparison between colchicine 0.005 mg/Kg/d or 0.01 mg/Kg/d arms and the placebo arm was carried out using the Bonferroni method to account for multiple arm comparisons, therefore Confidence Intervals (CI) are set at 97.5% and p value are considered statistically significant if <0.025. We did not apply any correction to the comparison of colchicine versus placebo arm, therefore CI are set at 95% and p value are considered statistically significant if <0.05.

Time point	Arm	Difference from baseline			MD	CI		p value
		n	mean	SD				
Week 4	Placebo	18	5.0	10.3	-			
	Colchicine 0.005 mg/kg/d	17	-2.2	9.0	-7.2	-15.13	0.78	0.049
	Colchicine 0.01 mg/kg/d	18	10.4	11.9	5.4	-2.45	13.23	0.130
	Colchicine	35	4.3	12.2	-0.7	-7.3	5.9	0.833
Week 12	Placebo	14	12.3	17.4	-			
	Colchicine 0.005 mg/kg/d	13	0.5	17.6	-11.8	-26.62	2.97	0.081
	Colchicine 0.01 mg/kg/d	16	16.8	16.5	4.5	-9.53	18.59	0.475
	Colchicine	29	9.5	18.7	-2.8	-14.4	8.8	0.640
Week 18	Placebo	15	12.5	15.5	-			
	Colchicine 0.005 mg/kg/d	12	4.6	15.6	-8.0	-21.56	5.66	0.198
	Colchicine 0.01 mg/kg/d	14	21.6	15.9	9.1	-3.95	22.17	0.126
	Colchicine	26	13.8	17.7	1.2	-9.5	12.0	0.823
	Placebo	12	16.5	15.5	-			
	Colchicine 0.005 mg/kg/d	10	9.8	21.1	-6.7	-23.74	10.34	0.385

Week 24	Colchicine 0.01 mg/kg/d	11	15.9	16.7	-0.6	-17.2	16.02	0.937
	Colchicine	21	13.0	18.7	-3.5	-16.0	9.0	0.588
Week 30	Placebo	13	20.9	18.6	-			
	Colchicine 0.005 mg/kg/d	9	11.7	19.4	-9.3	-27.16	8.65	0.256
	Colchicine 0.01 mg/kg/d	11	21.6	17.4	0.7	-16.2	17.63	0.925
	Colchicine	20	17.2	18.5	-3.8	-16.7	9.2	0.572
Week 42	Placebo	6	14.5	15.7	-			
	Colchicine 0.005 mg/kg/d	6	21.3	29.8	6.8	-22.59	36.26	0.609
	Colchicine 0.01 mg/kg/d	9	20.0	21.3	5.5	-21.36	32.36	0.652
	Colchicine	15	20.5	24.0	6.0	-14.9	27.0	0.579
Week 54	Placebo	7	28.1	18.6	-			
	Colchicine 0.005 mg/kg/d	7	25.0	23.1	-3.1	-30.87	24.58	0.802
	Colchicine 0.01 mg/kg/d	8	26.9	26.4	-1.3	-28.11	25.58	0.917
	Colchicine	15	26.0	24.1	-2.1	-22.4	18.1	0.838

SD = standard difference; MD = mean difference; CI = confidence interval.

Supplementary Table 11. ALSAQ40 administered at different time points in patients treated with colchicine and placebo.

Mean absolute changes from baseline to week 8-30-54 are showed for each treatment group and comparison were performed using linear regression models that include indicator variables for treatment arms as the independent variables. Comparison between colchicine 0.005 mg/Kg/d or 0.01 mg/Kg/d arms and the placebo arm was carried out using the Bonferroni method to account for multiple arm comparisons, therefore Confidence Intervals (CI) are set at 97.5% and p value are considered statistically significant if <0.025. We did not apply any correction to the comparison of colchicine versus placebo arm, therefore CI are set at 95% and p value are considered statistically significant if <0.05.

**ALSAQ40 domains**	**Time points**	**Arm**	**Absolute change From baseline**						
			**n**	**mean**	**SD**	**MD**	**CI**		**P Value**
Total % score	Week 8	Placebo	18	-6.15	6.82				
		Colchicine 0.005 mg/kg/d	17	0.07	5.80	6.22	1.37	11.07	**0.01**
		Colchicine 0.01 mg/kg/d	16	-4.06	6.53	2.08	-2.85	7.01	0.35
		Colchicine	33	-1.93	6.42	4.21	-0.09	8.52	**0.03**
	Week 30	Placebo	15	-15.17	11.04				
		Colchicine 0.005 mg/kg/d	15	-8.75	11.50	6.42	-2.61	15.44	0.12
		Colchicine 0.01 mg/kg/d	14	-12.95	10.46	2.22	-6.96	11.40	0.59
		Colchicine	29	-10.78	11.02	4.39	-3.47	12.25	0.22
	Week 54	Placebo	10	-18.81	8.46				
		Colchicine 0.005 mg/kg/d	11	-16.88	13.75	1.94	-9.02	12.89	0.69
		Colchicine 0.01 mg/kg/d	9	-22.15	10.27	-3.34	-14.86	8.18	0.52

		Colchicine	20	-19.25	12.29	-0.44	-10.16	9.29	0.92
Physical mobility % score	Week 8	Placebo	18	-11.25	18.59				
		Colchicine 0.005 mg/kg/d	17	3.82	11.96	15.07	4.47	25.68	**0.00**
		Colchicine 0.01 mg/kg/d	16	-1.41	9.04	9.84	-0.93	20.62	0.05
		Colchicine	33	1.29	10.81	12.54	3.34	21.74	**0.00**
	Week 30	Placebo	15	-11.50	16.92				
		Colchicine 0.005 mg/kg/d	15	-12.50	20.98	-1.00	-16.06	14.06	0.88
		Colchicine 0.01 mg/kg/d	14	-11.96	16.9	-0.46	-15.79	14.87	0.95
		Colchicine	29	-12.24	18.78	-0.74	-13.70	12.22	0.90
	Week 54	Placebo	10	-21.00	17.72				
		Colchicine 0.005 mg/kg/d	11	-16.82	18.34	4.18	-12.80	21.16	0.59
		Colchicine 0.01 mg/kg/d	9	-20.83	15.51	0.17	-17.69	18.02	0.98
		Colchicine	20	-18.63	16.81	2.38	-12.48	17.23	0.72
ADL and independence % score	Week 8	Placebo	18	-10.28	16.65				
		Colchicine 0.005 mg/kg/d	17	-2.21	13.23	8.07	-2.18	18.32	0.08
		Colchicine 0.01 mg/kg/d	16	-5.16	9.20	5.12	-5.29	15.54	0.28
		Colchicine	33	-3.64	11.37	6.64	-2.18	15.47	0.10
	Week 30	Placebo	15	-19.50	23.17				
		Colchicine 0.005 mg/kg/d	15	-8.67	18.78	10.83	-5.01	26.68	0.13
		Colchicine 0.01 mg/kg/d	14	-14.82	14.98	4.68	-11.45	20.81	0.52
		Colchicine	29	-11.64	17.04	7.86	-5.90	21.62	0.21
	Week	Placebo	10	-29.75	23.20				

	54	Colchicine 0.005 mg/kg/d	11	-26.82	19.37	2.93	-16.24	22.11	0.73
		Colchicine 0.01 mg/kg/d	9	-22.22	14.81	7.53	-12.64	27.69	0.41
		Colchicine	20	-24.75	17.19	5.00	-11.77	21.77	0.51
Eating and drinking % score	Week 8	Placebo	18	-4.63	14.35				
		Colchicine 0.005 mg/kg/d	17	-5.39	17.66	-0.76	-12.34	10.82	0.88
		Colchicine 0.01 mg/kg/d	16	-2.08	13.44	2.55	-9.22	14.31	0.63
		Colchicine	33	-3.79	15.60	0.84	-9.13	10.81	0.85
	Week 30	Placebo	15	-15.00	21.41				
		Colchicine 0.005 mg/kg/d	15	-13.89	29.99	1.11	-19.27	21.49	0.90
		Colchicine 0.01 mg/kg/d	14	-14.88	22.21	0.12	-20.62	20.86	0.99
		Colchicine	29	-14.37	26.06	0.63	-16.91	18.17	0.94
	Week 54	Placebo	10	-11.67	24.91				
		Colchicine 0.005 mg/kg/d	11	-9.85	20.35	1.82	-19.39	23.02	0.85
		Colchicine 0.01 mg/kg/d	9	-20.37	19.14	-8.70	-31.00	13.59	0.39
		Colchicine	20	-14.58	20.03	-2.92	-21.77	15.94	0.73
Communication % score	Week 8	Placebo	18	-4.17	19.30				
		Colchicine 0.005 mg/kg/d	17	-2.10	10.94	2.07	-9.28	13.41	0.69
		Colchicine 0.01 mg/kg/d	16	-6.47	12.92	-2.31	-13.83	9.22	0.66
		Colchicine	33	-4.22	11.96	-0.05	-9.85	9.75	0.99
	Week 30	Placebo	15	-19.52	22.13				
		Colchicine 0.005 mg/kg/d	15	-10.71	13.36	8.81	-5.76	23.38	0.18
		Colchicine 0.01 mg/kg/d	14	-16.07	16.74	3.45	-11.38	18.28	0.60

		Colchicine	29	-13.30	15.06	6.22	-6.42	18.86	0.28
	Week 54	Placebo	10	-18.21	24.54				
		Colchicine 0.005 mg/kg/d	11	-23.05	27.96	-4.84	-30.43	20.76	0.68
		Colchicine 0.01 mg/kg/d	9	-27.38	25.51	-9.17	-36.08	17.75	0.45
		Colchicine	20	-25.00	26.27	-6.79	-29.12	15.55	0.50
Emotional functioning % score	Week 8	Placebo	18	1.25	16.98				
		Colchicine 0.005 mg/kg/d	17	1.76	13.13	0.51	-10.20	11.23	0.91
		Colchicine 0.01 mg/kg/d	16	-4.53	11.34	-5.78	-16.67	5.10	0.24
		Colchicine	33	-1.29	12.52	-2.54	-11.88	6.81	0.55
	Week 30	Placebo	15	-11.50	18.32				
		Colchicine 0.005 mg/kg/d	15	-2.17	8.44	9.33	-1.97	20.63	0.07
		Colchicine 0.01 mg/kg/d	14	-9.29	12.76	2.21	-9.28	13.71	0.67
		Colchicine	29	-5.60	11.15	5.90	-4.05	15.85	0.19
	Week 54	Placebo	10	-8.25	16.63				
		Colchicine 0.005 mg/kg/d	11	-4.77	28.18	3.48	-18.18	25.13	0.72
		Colchicine 0.01 mg/kg/d	9	-20.28	18.60	-12.03	-34.80	10.74	0.25
		Colchicine	20	-11.75	25.03	-3.50	-23.18	16.18	0.69

SD = standard error; MD = mean difference; CI = confidence interval.

Supplementary Table 12: Changes in clinical outcome measures during and after treatment across treatment arms

Average monthly variations during and after treatment for the placebo group, as well as the comparisons between arms, are shown. Comparisons were performed using segmented repeated measures linear mixed models. Two segments of time were analyzed: during the treatment (after baseline and up to week 30), and after the treatment (after week 30). The dependent variables were the raw measurements of the outcomes, whereas the independent variables were: arm, time (months from baseline) x period (during or after treatment) interaction, and arm x time x period interaction. A random intercept term was also used to account for repeated measurements over the same individual, as well as a random slope term was used to account for individual linear variations over time. Random intercept and random slope terms were kept in the model if they improved the overall goodness-of-fit of the model. Comparison between colchicine 0.005 mg/kg/d or 0.01 mg/kg/d arms and the placebo arm was carried out using the Bonferroni method to account for multiple arm comparisons. We did not apply any correction to the comparison of Colchicine versus placebo arm.

Outcome	Time		Arm	MD	95% CI		p.value
ALSAQ40 Total_score	During treatment	Monthly variation	Placebo	3,47	-16,63	20,01	0,00
	After treatment	Monthly variation	Placebo	2,92	-7,56	29,11	0,00
	During treatment	Comparison with Placebo (monthly variation)	Co0005mg	-1,19	2,38	4,56	0,14
	During treatment		Co001mg	-0,40	1,98	3,88	0,63
	During treatment		Colchicine	-0,81	2,37	4,57	0,25
	After treatment		Co0005mg	-0,03	-2,74	0,35	0,97
	After treatment		Co001mg	0,33	-1,97	1,17	0,63
	After treatment		Colchicine	0,14	1,98	3,90	0,80
ALSAQ40 subscale: communication	During treatment	Monthly variation	Placebo	0,71	-3,99	7,39	0,00
	After treatment	Monthly variation	Placebo	0,58	-4,14	7,25	0,00
	During treatment	Comparison with Placebo (monthly variation)	Co0005mg	-0,24	0,36	1,06	0,36
	During treatment		Co001mg	0,02	0,27	0,89	0,93
	During treatment		Colchicine	-0,11	0,36	1,07	0,62
	After treatment		Co0005mg	0,02	-0,74	0,26	0,94
	After treatment		Co001mg	0,18	-0,48	0,53	0,43
	After treatment		Colchicine	0,10	0,27	0,89	0,62
ALSAQ40 subscale: eat_drink	During treatment	Monthly variation	Placebo	0,26	-1,30	2,56	0,01
	After treatment	Monthly variation	Placebo	0,20	-1,07	2,80	0,03
	During treatment	Comparison with Placebo (monthly variation)	Co0005mg	0,00	0,07	0,45	1,00
	During treatment		Co001mg	0,07	0,03	0,38	0,64
	During treatment		Colchicine	0,03	0,06	0,45	0,79

	After treatment		Co0005mg	0,03	-0,27	0,28	0,80
	After treatment		Co001mg	0,15	-0,21	0,35	0,26
	After treatment		Colchicine	0,09	0,03	0,39	0,43
ALSAQ40 subscale: emotion	During treatment	Monthly variation	Placebo	0,66	-4,71	7,09	0,01
	After treatment		Placebo	0,26	-4,52	7,30	0,16
	During treatment	Comparison with Placebo (monthly variation)	Co0005mg	-0,42	0,21	1,11	0,21
	During treatment		Co001mg	-0,20	-0,10	0,61	0,55
	During treatment		Colchicine	-0,32	0,20	1,11	0,28
	After treatment		Co0005mg	0,04	-1,06	0,22	0,89
	After treatment		Co001mg	0,29	-0,85	0,44	0,27
	After treatment		Colchicine	0,16	-0,11	0,61	0,49
ALSAQ40 subscale: independence	During treatment	Monthly variation	Placebo	1,12	-9,94	7,33	0,00
	After treatment		Placebo	1,08	-4,95	12,34	0,00
	During treatment	Comparison with Placebo (monthly variation)	Co0005mg	-0,58	0,63	1,60	0,11
	During treatment		Co001mg	-0,28	0,69	1,47	0,44
	During treatment		Colchicine	-0,44	0,63	1,61	0,16
	After treatment		Co0005mg	-0,08	-1,26	0,11	0,75
	After treatment		Co001mg	-0,27	-0,98	0,41	0,34
	After treatment		Colchicine	-0,16	0,69	1,46	0,49
ALSAQ40 subscale: physical_mob	During treatment	Monthly variation	Placebo	0,62	-7,78	6,50	0,01
	After treatment		Placebo	0,60	-3,96	10,32	0,00
	During treatment	Comparison with Placebo (monthly variation)	Co0005mg	0,19	0,13	1,09	0,59
	During treatment		Co001mg	0,07	0,26	0,93	0,84
	During treatment		Colchicine	0,13	0,13	1,10	0,67
	After treatment		Co0005mg	0,14	-0,48	0,87	0,57
	After treatment		Co001mg	0,14	-0,61	0,76	0,59
	After treatment		Colchicine	0,13	0,26	0,94	0,53
FVC	During treatment	Monthly variation	Placebo	-3,80	-5,36	-2,28	0,00
	After treatment		Placebo	-4,04	-5,60	-2,54	0,00
	During treatment	Comparison with Placebo (monthly variation)	Co0005mg	1,47	-0,75	3,69	0,21
	During treatment		Co001mg	-0,26	-2,42	1,90	0,82
	During treatment		Colchicine	0,58	-1,36	2,52	0,56
	After treatment		Co0005mg	0,92	-1,24	3,09	0,41
	After treatment		Co001mg	0,35	-1,75	2,45	0,75

After treatment	Colchicine	0,64	-1,24	2,55	0,51

Supplementary Table 13: Changes in mRNA contents in PBMC comparing baseline and week 30.

Gene expression data (mRNA) are presented in the form 2 ^ (- ΔΔCt), where for each gene and for each patient the ΔΔCt is calculated as the difference between ΔCt at time 1 (Week 30) and ΔCt at time 0 (Baseline). Since the two temporal measures were summarized in a single value, the analysis for repeated measures was not performed. Instead, Mann-Whitney tests were performed to see if the arms of the study had different mean values of 2 ^ (- ΔΔCt) for each gene.

HSPB8 expression was analyzed in all samples, but its expression was below the detection level in all groups, included those treated with colchicine. Alternatively, we included the analysis of another member of the HSPB family, which also interacts with BAG3, the HSPB1. HSPB1 levels were detectable in the assay, but no changes were found in all conditions tested. Since the levels of TFEB, the master regulator of autophagy-related genes (including ATGs), were found unchanged in all condition tested, we did not evaluate the expression levels of ATGs. However, to exclude alternative regulation, we also tested the expression of TFE3 (which overlaps the regulation mediated by TFEB in the activation of ATGs), but its expression was below the detection level in PBMC.

Comparison between colchicine 0.005 mg/kg/d or 0.01 mg/kg/d arms and the placebo arm was carried out using the Bonferroni method to account for multiple arm comparisons (p value are considered statistically significant if <0.025). We did not apply any correction to the comparison of colchicine versus placebo arm, therefore p value are considered statistically significant if <0.05.

	**Colchicine 0.005 mg/kg/d**			**Colchicine 0.01 mg/kg/d**			**Colchicine**			**Placebo**		
Gene	N	Mean	SD	N	Mean	SD	N	Mean	SD	N	Mean	SD
HSPB1	9	1.536	0.873	9	2.369	2.212	18	1.953	1.687	9	1.811	1.375
BAG3	9	2.038	1.306	9	1.455	1.246	18	1.747	1.274	9	1.164	0.461
BAG1	8	1.555	1.037	9	1.686	1.391	17	1.625	1.201	7	0.957	0.832
HSF1	9	1.595	1.134	9	1.767	1.353	18	1.681	1.214	8	1.419	0.768
TFEB	9	1.772	2.671	9	2.397	2.948	18	2.084	2.748	9	1.329	0.735
SQSTM1 p62	9	0.900	0.449	9	1.919	1.592	18	1.409	1.250	9	0.736	0.337

MAP1LC3B	9	1.097	0.588	9	2.461	2.548	18	1.779	1.926	9	0.882	0.503
HSPA6	9	2.726	2.949	9	24.985	36.603	18	13.856	27.672	9	1.275	1.092

SD = standard deviation.

	Wilcoxon-Mann-Whitney p-values		
	**Colchicine 0.005 mg/kg/d vs Placebo**	**Colchicine 0.01 mg/kg/d vs Placebo**	**Colchicine Vs placebo**
HSPB1	0.666	0.730	0.980
BAG3	0.258	0.931	0.561
BAG1	0.121	0.252	0.114
HSF1	0.963	0.963	0.935
TFEB	0.546	1.000	0.705
SQSTM1 p62	0.489	0.063	0.131
MAP1LC3B	0.605	0.077	0.176
HSPA6	0.730	0.040	0.160

Supplementary Table 14: Changes in mRNA contents in PMBC comparing baseline and week 54.

Supplementary Table 14 shows changes in mRNA contents in PMBC comparing baseline and week 54 on a limited number of samples that were available for three-time points examinations. There were no significant differences between treatment and placebo arm as far as these genes/proteins are concerned at this time point (week 54) with respect to baseline.

Comparison between colchicine 0.005 mg/kg/d or 0.01 mg/kg/d arms and the placebo arm was carried out using the Bonferroni method to account for multiple arm comparisons (p value are considered statistically significant if <0.025). We did not apply any correction to the comparison of colchicine versus placebo arm, therefore p value are considered statistically significant if <0.05.

	**Colchicine 0.005 mg/kg/d**			**Colchicine 0.01 mg/kg/d**			**Colchicine**			**Placebo**		
Gene	N	Mean	SD	N	Mean	SD	N	Mean	SD	N	Mean	SD
HSPB1	6	0.807	0.614	4	2.226	1.266	10	1.977	1.434	6	1.082	0.538
BAG3	6	1.187	0.954	4	1.674	1.088	10	1.474	1.386	6	0.777	0.737
BAG1	6	0.939	0.738	4	1.255	0.982	10	2.308	2.375	6	0.682	0.308
HSF1	6	1.338	0.822	4	1.705	1.097	10	1.485	0.902	6	1.015	0.391
TFEB	6	1.845	1.672	4	0.917	0.639	10	1.375	1.131	6	1.556	1.248
SQSTM1 p62	6	1.980	1.225	4	1.972	1.916	10	1.066	0.807	6	1.166	1.106
MAP1LC3B	5	2.284	2.084	4	2.339	3.042	9	1.382	0.982	6	0.928	0.753
HSPA6	6	2.703	3.730	4	2.095	2.127	10	2.460	3.055	6	0.953	0.645

SD = standard deviation.

	Wilcoxon-Mann-Whitney p-values		
	**Colchicine 0.005 mg/kg/d vs Placebo**	**Colchicine 0.01 mg/kg/d vs Placebo**	**Colchicine vs Placebo**
HSPB1	0.485	0.171	0.875
BAG3	0.589	0.257	0.313
BAG1	0.394	0.610	0.368
HSF1	0.589	0.352	0.368
TFEB	1.000	0.476	0.713
SQSTM1 p62	0.240	0.476	0.220
MAP1LC3B	0.429	0.476	0.328
HSPA6	1.000	0.762	0.875

Supplementary Table 15: Changes in insoluble TDP43 protein levels in PBMC at baseline (T0) and week 30 (T1) in placebo and colchicine-treated groups.

Comparison between colchicine 0.005 mg/kg/d or 0.01 mg/kg/d arms and the placebo arm was carried out using the Bonferroni method to account for multiple arm comparisons (p value are considered statistically significant if <0.025). We did not apply any correction to the comparison of colchicine versus placebo arm, therefore p value are considered statistically significant if <0.05.

	T0 - Mean (SD)				T1 - Mean (SD)			
TDP43	**Colchicine 0.005 mg/kg/d (N=5)**	**Colchicine 0.01 mg/kg/d (N=4)**	**Colchicine (N=9)**	**Placebo (N=4)**	**Colchicine 0.005 mg/kg/d (N=5)**	**Colchicine 0.01 mg/kg/d (N=4)**	**Colchicine (N=9)**	**Placebo (N=4)**
	1.00 (0.020)	0.998 (0.032)	0.999 (0.024)	1.00 (0.060)	0.366 (0.338)	1.322 (1.271)	0.791 (0.958)	2.684 (2.001)

	Mean difference from T1 and T0 (SD)				p-value		
	**Colchicine 0.005 mg/kg/d (N=5)**	**Colchicine 0.01 mg/kg/d (N=4)**	**Colchicine (N=9)**	**Placebo (N=4)**	**Colchicine 0.005 mg/kg/d vs Placebo**	**Colchicine 0.01 mg/kg/d vs Placebo**	**Colchicine vs Placebo**
TDP43	-0.634 (0.331)	0.325 (1.245)	-0.208 (0.944)	1.684 (2.035)	0.031	0.177	**0.038**

SD = standard deviation.

Supplementary Table 16: Quantity of DRiPs inside SGs at baseline (T0) and week 30 (T1) across treatment arms. Comparison between colchicine 0.005 mg/kg/d or 0.01 mg/kg/d arms and the placebo arm was carried out using the Bonferroni method to account for multiple arm comparisons (p value are considered statistically significant if <0.025). We did not apply any correction to the comparison of colchicine versus placebo arm, therefore p value are considered statistically significant if <0.05.

	T0 - Mean (SD)				T1 - Mean (SD)			
	**Colchicine 0.005 mg/kg/d (N=13)**	**Colchicine 0.01 mg/kg/d (N=15)**	**Colchicine (N=26)**	**Placebo (N=11)**	**Colchicine 0.005 mg/kg/d (N=3)**	**Colchicine 0.01 mg/kg/d (N=7)**	**Colchicine (N=10)**	**Placebo (N=5)**
DRiP	1.280 (0.116)	1.273 (0.228)	1.276 (0.183)	1.275 (0.148)	1.221 (0.166)	1.311 (0.162)	1.277 (0.158)	1.350 (0.203)
DRiP 0-1.5 (%)	85.548 (5.768)	84.933 (11.416)	85.164 (9.169)	85.601 (8.040)	89.888 (5.772)	83.26 (7.741)	85.750 (7.452)	81.766 (10.534)
DRiP 1.5-3.0 (%)	13.037 (4.786)	13.769 (10.025)	13.494 (8.001)	13.566 (7.441)	9.863 (5.700)	15.231 (6.834)	13.218 (6.610)	16.885 (9.194)
DRiP >3.0 (%)	1.237 (1.017)	1.284 (1.439)	1.266 (1.216)	0.788 (0.792)	0.226 (0.060)	1.452 (0.912)	0.992 (0.938)	1.321 (1.341)
	Mean difference from T0 and T1				p-value			
	**Colchicine 0.005 mg/kg/d (N=3)**	**Colchicine 0.01 mg/kg/d (N=5)**	**Colchicine (N=8)**	**Placebo (N=4)**	**Colchicine 0.005 mg/kg/d vs Placebo**	**Colchicine 0.01 mg/kg/d vs Placebo**	**Colchicine mg/kg/d vs Placebo**	
DRiP	-0.060 (0.270)	0.038 (0.246)	0.001 (0.241)	0.075 (0.332)	0.857	0.905	0.808	
DRiP 0-1.5 (%)	4.340 (11.335)	-1.673 (11.191)	0.582 (10.861)	-3.835 (17.935)	0.400	1.000	0.683	

DRiP 1.5-3.0 (%)	-3.174 (10.144)	1.463 (10.021)	-0.276 (9.620)	3.318 (16.104)	0.400	1.000	0.683
DRiP <3.0 (%)	-1.011 (1.068)	0.168 (1.245)	-0.274 (1.258)	0.533 (1.915)	0.400	0.905	0.570

SD = standard deviation.

Supplementary Table 17: Exosomes (EXOs) and microvescicles (MVs) plasma concentration across treatment arms at baseline (T0) and week 30 (T1).

Comparison between colchicine 0.005 mg/kg/d or 0.01 mg/kg/d arms and the placebo arm was carried out using the Bonferroni method to account for multiple arm comparisons (p value are considered statistically significant if <0.025). We did not apply any correction to the comparison of colchicine versus placebo arm, therefore p value are considered statistically significant if <0.05.

	T0 - Mean (SD)				T1 - Mean (SD)			
	**Colchicine 0.005 mg/kg/d (N=8)**	**Colchicine 0.01 mg/kg/d (N=8)**	**Colchicine (N=16)**	**Placebo (N=7)**	**Colchicine 0.005 mg/kg/d (N=8)**	**Colchicine 0.01 mg/kg/d (N=8)**	**Colchicine (N=16)**	**Placebo (N=7)**
EXOs	-0.03 (0.964)	0.137 (1.417)	0.053 (1.174)	-0.172 (1.116)	-0.595 (0.665)	-0.202 (0.656)	-0.398 (0.669)	-0.05 (0.778)
MVs	-0.348 (0.618)	-0.222 (0.85)	-0.285 (0.721)	-0.025 (1.355)	-0.416 (0.927)	0.283 (1.374)	-0.067 (1.188)	0.065 (0.732)

	Mean difference from T0 and T1				p-value		
	**Colchicine 0.005 mg/kg/d (N=8)**	**Colchicine 0.01 mg/kg/d (N=8)**	**Colchicine (N=16)**	**Placebo (N=7)**	**Colchicine 0.005 mg/kg/dvs Placebo**	**Colchicine 0.01 mg/kg/d vs Placebo**	**Colchicine mg/kg/dvs Placebo**
EXOs	-0.565 (1.350)	-0.339 (1.234)	-0.452 (1.255)	0.122 (0.586)	0.281	0.613	0.341
MVs	-0.068 (0.578)	0.505 (1.196)	0.219 (0.954)	0.091 (1.446)	0.397	0.613	0.871

SD = standard deviation.

Supplementary Table 18. Changes of biomarkers of neuroinflammation and neurodegeneration across treatment arms between baseline (T0) and week 30 (T1) and week 54 (T2) in plasma. Comparison between colchicine 0.005 mg/Kg/d or 0.01 mg/Kg/d arms and the placebo arm was carried out using the Bonferroni method to account for multiple arm comparisons, therefore Confidence Intervals (CI) are set at 97.5% and p value are considered statistically significant if <0.025. We did not apply any correction to the comparison of colchicine versus placebo arm, therefore CI are set at 95% and p value are considered statistically significant if <0.05.

**N. of samples**	**Colchicine 0.005 mg/kg/d - T0**	**Colchicine 0.01 mg/kg/d–T0**	**Placebo – T0**	**Colchicine 0.005 mg/kg/d –T1**	**Colchicine 0.01 mg/kg/d –T1**	**Placebo – T1**	**Colchicine 0.005 mg/kg/d –T2**	**Colchicine 0.01 mg/kg/d –T2**	**Placebo – T2**
NFL	18	18	18	13	14	14	8	7	8
IL17A	15*	13*	13*	10**	11**	9**	7***	6***	5***
IL18	18	18	18	12**	14	14	8	7	8
IL18BP	18	18	18	13	14	14	8	7	8
MCP1	18	18	18	13	14	14	8	7	8

*54 samples collected: the missing values for IL-17A are related to samples with concentrations below the detection limit.

**41 samples collected: the missing values for IL-17A and IL-18 are related to samples with concentrations below the detection limit.

***23 samples collected: the missing values for IL-17A are related to samples with concentrations below the detection limit.

Biomarker	Arm	Mean (SD) - T0	Mean (SD) - T1	Difference, mean (SD)	MD	lower CI	higher CI	P value
NFL	Placebo	111,68 (92,14)	115,48 (86,67)	3,8 (57,55)				
	Colc. 0.005 mg/kg/d	78,72 (38,22)	76,65 (36,56)	-2,07 (17,33)	-5,86	-39,73	28,00	0,70
	Colc. 0.01 mg/kg/d	94,49 (66,4)	108,44 (83,18)	13,95 (30,15)	10,15	-23,08	43,39	0,50
	Colchicine	86,9 (54,25)	93,14 (65,87)	6,24 (25,68)	2,44	-26,56	31,44	0,85
IL17A	Placebo	0,27 (0,26)	0,7 (1,31)	0,43 (1,12)				
	Colc. 0.005 mg/kg/d	0,15 (0,15)	0,22 (0,2)	0,06 (0,11)	-0,37	-1,02	0,29	0,22
	Colc. 0.01 mg/kg/d	0,17 (0,17)	0,25 (0,28)	0,09 (0,27)	-0,35	-1,00	0,31	0,25
	Colchicine	0,16 (0,16)	0,24 (0,23)	0,07 (0,2)	-0,36	-0,92	0,21	0,17
IL18	Placebo	387,41 (191,38)	395 (220,76)	7,59 (89,96)				
	Colc. 0.005 mg/kg/d	278,59 (112,24)	357,27 (219,71)	78,68 (161,29)	71,09	-31,94	174,12	0,13
	Colc. 0.01 mg/kg/d	402,31 (175,22)	394,2 (164,53)	-8,11 (93,54)	- 15,70	- 114,69	83,28	0,72
	Colchicine	345,21 (159,57)	377,15 (188,86)	31,94 (133,95)	24,36	-65,34	114,06	0,55
IL18BP	Placebo	1,69 (0,34)	1,69 (0,28)	0,01 (0,24)				

	Colc. 0.005 mg/kg/d	1,76 (0,32)	1,72 (0,3)	-0,05 (0,32)	-0,06	-0,30	0,19	0,61
	Colc. 0.01 mg/kg/d	1,63 (0,29)	1,67 (0,26)	0,03 (0,27)	0,02	-0,21	0,26	0,82
	Colchicine	1,7 (0,3)	1,69 (0,27)	-0,01 (0,29)	-0,01	-0,22	0,19	0,88
MCP1	Placebo	36,69 (15,72)	36,09 (10,71)	-0,61 (9,41)				
	Colc. 0.005 mg/kg/d	32,77 (13,26)	41,95 (26,38)	9,18 (16,23)	9,79	-0,22	19,81	0,03
	Colc. 0.01 mg/kg/d	37,64 (15,98)	41,59 (13,79)	3,95 (7,85)	4,56	-5,27	14,39	0,30
	Colchicine	35,29 (14,66)	41,76 (20,4)	6,47 (12,63)	7,08	-1,52	15,69	0,07

**Biomarker**	**Arm**	**Mean (SD) - T0**	**Mean (SD) – T2**	**Difference, mean (SD)**	**MD**	**lower CI**	**higher CI**	**P value**
NFL	Placebo	63,05 (30,41)	69,82 (24,62)	6,77 (21,14)				
	Colc. 0.005 mg/kg/d	79,69 (39,08)	62,75 (27,55)	-16,94 (23,7)	-23,71	-55,35	7,92	0,11
	Colc. 0.01 mg/kg/d	68,92 (43,99)	65,81 (31,5)	-3,11 (38,47)	-9,89	-42,63	22,86	0,51
	Colchicine	74,66 (40,3)	64,18 (28,41)	-10,49 (31,08)	-17,26	-44,89	10,37	0,18
IL17A	Placebo	0,28 (0,28)	0,24 (0,23)	-0,04 (0,29)				

	Colc. 0.005 mg/kg/d	0,17 (0,14)	0,29 (0,15)	0,12 (0,04)	0,16	-0,29	0,60	0,44
	Colc. 0.01 mg/kg/d	0,23 (0,2)	0,51 (0,36)	0,29 (0,4)	0,32	-0,10	0,75	0,12
	Colchicine	0,2 (0,17)	0,41 (0,29)	0,21 (0,29)	0,25	-0,14	0,63	0,17
IL18	Placebo	469,54 (207,15)	440,18 (213,74)	-29,36 (81,35)				
	Colc. 0.005 mg/kg/d	306,38 (112,39)	487,54 (609,23)	181,16 (584,12)	210,52	-186,96	608,00	0,25
	Colc. 0.01 mg/kg/d	393,17 (174,09)	415,38 (219,29)	22,21 (116,33)	51,57	-359,87	463,00	0,78
	Colchicine	346,88 (145,99)	453,87 (455,61)	106,98 (427,94)	136,34	-209,61	482,30	0,39
IL18BP	Placebo	1,68 (0,17)	1,73 (0,16)	0,05 (0,16)				
	Colc. 0.005 mg/kg/d	1,72 (0,34)	1,65 (0,27)	-0,06 (0,28)	-0,11	-0,38	0,15	0,36
	Colc. 0.01 mg/kg/d	1,67 (0,28)	1,75 (0,24)	0,08 (0,26)	0,03	-0,25	0,30	0,82
	Colchicine	1,7 (0,3)	1,7 (0,25)	0 (0,27)	-0,05	-0,28	0,19	0,66
MCP1	Placebo	30,01 (7,94)	35,5 (9,33)	5,49 (9,44)				
	Colc. 0.005 mg/kg/d	35,4 (16,13)	37,04 (17,78)	1,65 (7,54)	-3,84	-19,72	12,03	0,59
	Colc. 0.01 mg/kg/d	42,81 (19,95)	54,41 (34,8)	11,6 (22,34)	6,11	-10,33	22,55	0,41
	Colchicine	38,85	45,15	6,29 (16,39)	0,80	-13,38	14,98	0,90

		(17,75)	(27,52)	

SD = standard deviation; MD = mean difference; CI = confidence interval

Supplementary Table 19. Changes of biomarkers of neuroinflammation and neurodegeneration across treatment arms between baseline (T0) and week 30 (T1) in CSF. Comparison between colchicine 0.005 mg/Kg/d or 0.01 mg/Kg/d arms and the placebo arm was carried out using the Bonferroni method to account for multiple arm comparisons, therefore Confidence Intervals (CI) are set at 97.5% and p value are considered statistically significant if <0.025. We did not apply any correction to the comparison of colchicine versus placebo arm, therefore CI are set at 95% and p value are considered statistically significant if <0.05.

N. of samples	Colchicine 0.005 mg/kg/d - T0	Colchicine 0.01 mg/kg/d– T0	Placebo – T0	Colchicine 0.005 mg/kg/d – T1	Colchicine 0.01 mg/kg /d –T1	Placebo – T1
NFL	15	17	16	10	8	8
pNFH	15	17	16	10	8	8
IL17A	10*	16*	12*	9**	7**	8
IL18	15	17	16	10	8	8
IL18BP	15	17	16	10	8	8
MCP1	15	17	16	10	8	8

*48 samples collected: the missing values for IL-17A are related to samples with concentrations below the detection limit.

**26 samples collected: the missing values for IL-17A are related to samples with concentrations below the detection limit.

Biomarker	Arm	Mean (SD) - T0	Mean (SD) - T1	Difference, mean (SD)	MD	lower CI	higher CI	p value
NFL	Placebo	6764,86 (6632,63)	8101,3 (4619,42)	1336,44 (2973,3)				
	Colc. 0.005 mg/kg/d	6076,38 (2717,14)	6504,48 (5540,61)	428,1 (4120,16)	-908,34	-4587,83	2771,15	0,59
	Colc. 0.01 mg/kg/d	7843,27 (4003,51)	6761,04 (3232,08)	-1082,23 (2203,31)	-2418,68	-6197,44	1360,08	0,17
	Colchicine	6907,86 (3395,6)	6625,21 (4465,07)	-282,64 (3348,96)	-1619,09	-4891,32	1653,15	0,28
IL17A	Placebo	0,35 (0,2)	0,45 (0,21)	0,11 (0,18)				
	Colc. 0.005 mg/kg/d	0,47 (0,1)	0,49 (0,09)	0,02 (0,16)	-0,09	-0,33	0,16	0,43
	Colc. 0.01 mg/kg/d	0,51 (0,05)	0,4 (0,19)	-0,11 (0,22)	-0,22	-0,45	0,02	0,05
	Colchicine	0,49 (0,08)	0,44 (0,15)	-0,05 (0,2)	-0,16	-0,36	0,05	0,11
IL18	Placebo	3,33 (1,73)	3,27 (1,13)	-0,06 (1,09)				
	Colc. 0.005 mg/kg/d	2,93 (1,41)	2,62 (1,35)	-0,3 (1,36)	-0,24	-1,59	1,10	0,69
	Colc. 0.01 mg/kg/d	3,29 (1,76)	3,85 (2,59)	0,56 (1,05)	0,62	-0,76	2,00	0,33
	Colchicine	3,1 (1,55)	3,2 (2,06)	0,1 (1,27)	0,16	-1,07	1,39	0,77
IL18BP	Placebo	4,28 (0,78)	4,23 (0,82)	-0,05 (0,47)				

	Colc. 0.005 mg/kg/d	4,13 (0,65)	4,2 (0,76)	0,06 (0,41)	0,11	-0,34	0,56	0,58
	Colc. 0.01 mg/kg/d	4,5 (0,65)	4,49 (0,74)	-0,01 (0,31)	0,04	-0,43	0,50	0,86
	Colchicine	4,31 (0,66)	4,33 (0,74)	0,03 (0,36)	0,08	-0,32	0,47	0,67
MCP1	Placebo	282,74 (54,91)	295,95 (74,72)	13,21 (40,26)				
	Colc. 0.005 mg/kg/d	266,35 (67,44)	294,91 (117,39)	28,56 (61,89)	15,34	-39,07	69,76	0,53
	Colc. 0.01 mg/kg/d	296,95 (80,84)	292,76 (56,59)	-4,19 (34,58)	-17,40	-73,29	38,48	0,49
	Colchicine	280,75 (73,36)	293,9 (91,06)	13,15 (52,17)	-0,07	-49,60	49,47	1,00
pNFH	Placebo	2,46 (1,38)	2,31 (1,24)	-0,15 (0,45)				
	Colc. 0.005 mg/kg/d	2,08 (1,25)	2 (1,38)	-0,08 (0,27)	0,07	-0,58	0,71	0,82
	Colc. 0.01 mg/kg/d	2,73 (1,16)	2,38 (0,94)	-0,35 (0,85)	-0,20	-0,87	0,46	0,50
	Colchicine	2,38 (1,22)	2,18 (1,18)	-0,21 (0,61)	-0,06	-0,64	0,51	0,81

SD = standard deviation; MD = mean difference; CI = confidence interval.

Supplementary Table 20: Mean difference from baseline to week 30 and 54 in CK, albumin, creatinine and vitamin D between colchicine and placebo arms.

Comparison between colchicine 0.005 mg/Kg/d or 0.01 mg/Kg/d arms and the placebo arm was carried out using the Bonferroni method to account for multiple arm comparisons, therefore Confidence Intervals (CI) are set at 97.5% and p value are considered statistically significant if <0.025. We did not apply any correction to the comparison of colchicine versus placebo arm, therefore CI are set at 95% and p value are considered statistically significant if <0.05.

	Timepoint	Arm	N	Mean	SD	MD	CI		p value
CK	Difference from baseline to week 30	Placebo	15	-43.647	338.063				
		Colchicine 0.005 mg/kg/d	12	46.500	408.434	90.147	-198.500	378.793	0.488
		Colchicine 0.01 mg/kg/d	14	18.193	242.789	61.840	-215.116	338.795	0.620
		Colchicine	26	31.258	322.892	74.904	-133.802	283.611	0.486
	Difference from baseline to week 54	Placebo	9	51.333	386.995				
		Colchicine 0.005 mg/kg/d	10	27.600	436.616	-23.733	-397.297	349.830	0.888
		Colchicine 0.01 mg/kg/d	8	-2.888	186.706	-54.221	-449.284	340.843	0.761
		Colchicine	18	14.050	339.883	-37.283	-321.848	247.281	0.799
Creatinine	Difference from	Placebo	15	0.041	0.209				
		Colchicine	12	0.110	0.322	0.069	-0.133	0.272	0.448

	baseline to week 30	0.005 mg/kg/d							
		Colchicine 0.01 mg/kg/d	14	0.084	0.158	0.044	-0.151	0.238	0.618
		Colchicine	26	0.096	0.242	0.055	-0.091	0.202	0.463
	Difference from baseline to week 54	Placebo	9	-0.004	0.201				
		Colchicine 0.005 mg/kg/d	10	0.162	0.333	0.166	-0.099	0.432	0.172
		Colchicine 0.01 mg/kg/d	8	0.011	0.196	0.016	-0.265	0.296	0.901
		Colchicine	18	0.095	0.284	0.099	-0.109	0.308	0.358
Albumin	Difference from baseline to week 30	Placebo	15	-0.073	0.392				
		Colchicine 0.005 mg/kg/d	10	-0.002	0.511	0.071	-0.281	0.423	0.652
		Colchicine 0.01 mg/kg/d	13	-0.218	0.236	-0.145	-0.472	0.181	0.326
		Colchicine	23	-0.124	0.386	-0.051	-0.304	0.202	0.695
	Difference from baseline to week 54	Placebo	8	-0.313	0.391				
		Colchicine 0.005 mg/kg/d	9	0.031	0.510	0.344	-0.098	0.786	0.096
		Colchicine 0.01 mg/kg/d	7	-0.187	0.226	0.125	-0.345	0.596	0.557
		Colchicine	16	-0.064	0.415	0.248	-0.097	0.594	0.173
Vitamin	Difference	Placebo	15	-2.373	12.909				

D	from baseline to week 30	Colchicine 0.005 mg/kg/d	10	4.340	12.796	6.713	-5.048	18.475	0.210
		Colchicine 0.01 mg/kg/d	11	-0.573	12.827	1.801	-9.636	13.237	0.726
		Colchicine	21	1.767	12.739	4.140	-4.347	12.627	0.346

SD = standard deviation; MD = mean difference; CI = confidence interval.

4. Supplementary Figures

Supplementary Figure 1. Individual rates of decline in ALSFRS-R total score of patients enrolled in co-ALS over the study. Individual rates of decline in Amyotrophic Lateral Sclerosis Functional Rating Scale-Revised (ALSFRS-R) total score (Intention-to-treat and Per-Protocol population) of patients enrolled in co-ALS over the study (baseline to week 54) based on treatment arm allocation: placebo (box A), colchicine 0.05 mg/kg/d (box B), colchicine 0.01 mg/kg/d (box C)

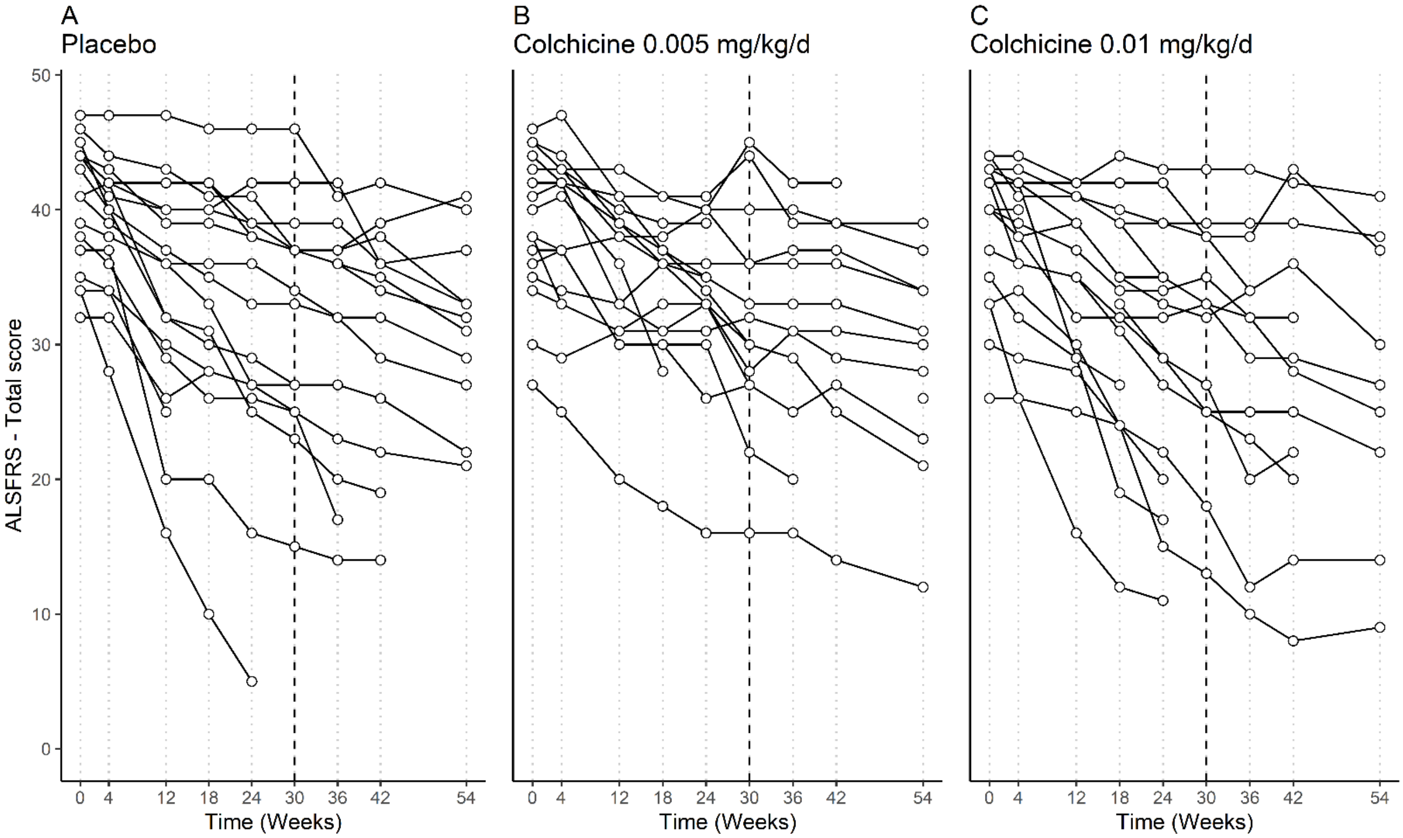

ALSFRS-R = Amyotrophic Lateral Sclerosis Functional Rating Scale-Revised

Supplementary Figure 2. Tracheostomy-free survival from baseline based on treatment arm allocation. Tracheostomy-free survival from baseline based on treatment arm allocation (blue = colchicine 0.005 mg/kg/d, yellow = colchicine 0.01 mg/kg/d, gray = placebo). Thick marks represent participants lost to follow-up. The number of participants at risk is displayed in the table.

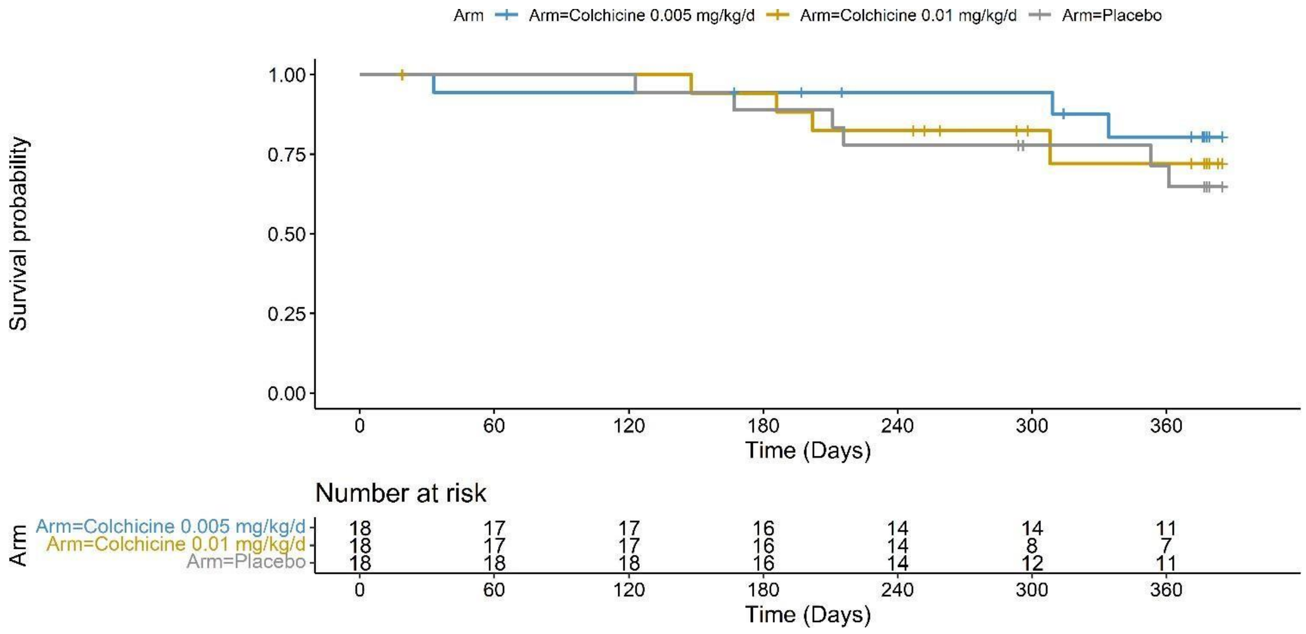

Supplementary Figure 3. Respiratory muscle function as assessed by forced vital capacity score decline from baseline to weeks 4, 12, 18, 24, 30, 42, and 54 across treatment arms.

Mean rates of decline in forced vital capacity (FVC) % (Intention-to-treat population) of patients enrolled in co-ALS over the study (baseline to week 54) based on treatment arm allocation (blue = colchicine 0.005 mg/kg/d, yellow = colchicine 0.01 mg/kg/d, gray = placebo).

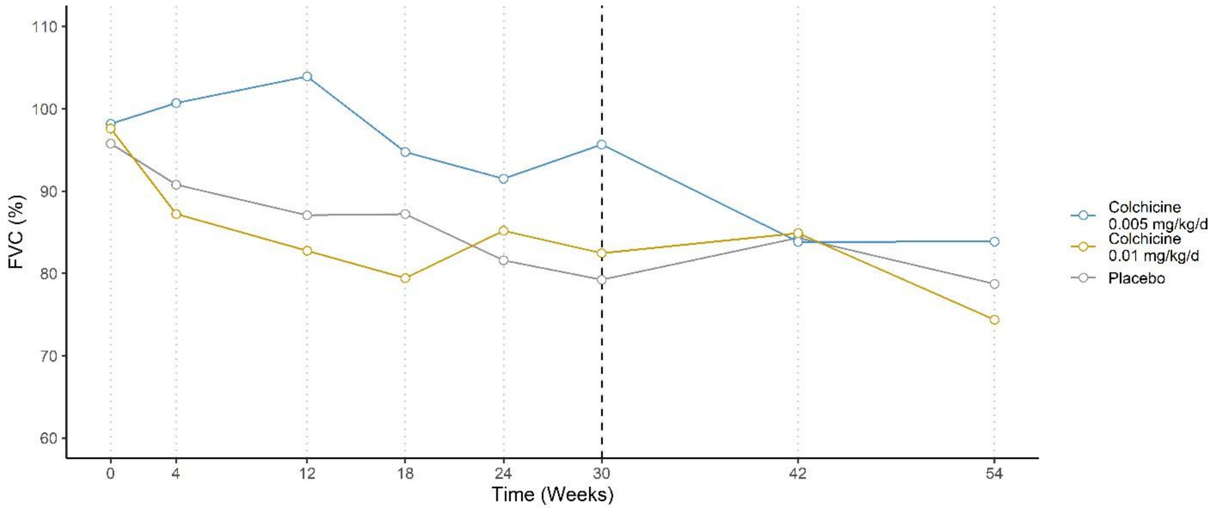

Time (Weeks)	0	4	12	18	24	30	42	54
Placebo	18	18	14	15	12	13	6	7
Colchicine 0.005 mg/kg/d	18	17	13	12	10	9	6	7
Colchicine 0.01 mg/kg/d	18	18	16	14	11	11	9	8

Supplementary Figure 4. Mean scores of ALSAQ40 from baseline to the end of the study across treatment arms. Panel **A** shows Amyotrophic Lateral Sclerosis Assessment Questionnaire ALSAQ40 mean total score from baseline to study end (red = colchicine 0.005 mg/kg/d, blue = colchicine 0.01 mg/kg/d, green= placebo; Intention-to-treat population). Panels **B** through **F** show the treatment-dependent mean scores of ALSAQ40 communication. emotional functioning. physical mobility. ADL and independence. and eating and drinking main questions, respectively, from baseline to study end.

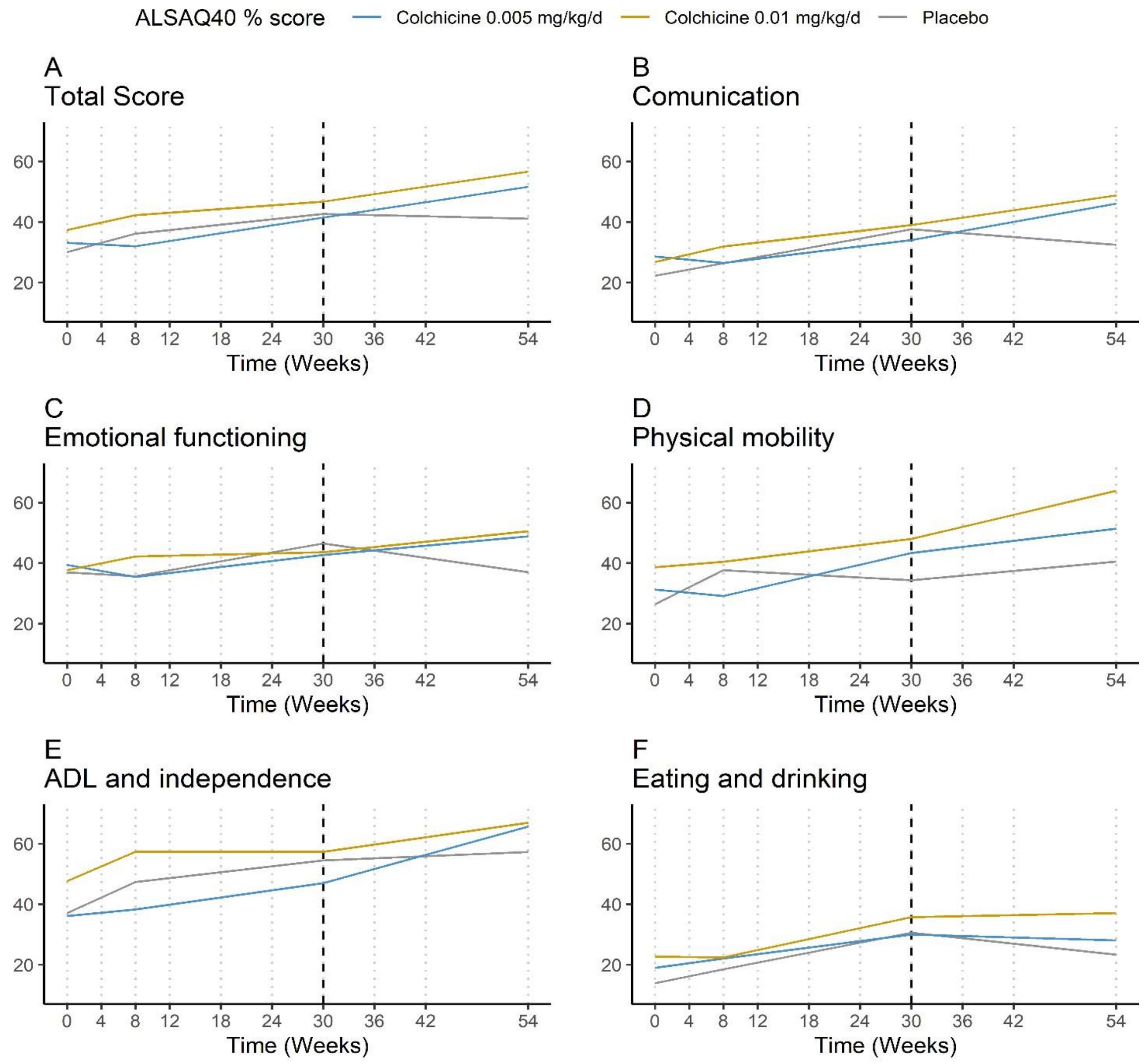

Number of observations

Time (Weeks)	0	54	30	54
Placebo	18	10	15	10
Colchicine 0.005 mg/kg/d	18	11	15	11
Colchicine 0.01 mg/kg/d	18	9	14	9

ALSAQ40 = Amyotrophic Lateral Sclerosis Assessment Questionnaire, ADL = Activity Daily Living

Supplementary Figure 5. Effects on autophagy related gene (HSPB1, BAG3, BAG1, HSF1, TFEB, SQSTM1 p62, MAP1LC3B, HSPA6) expression by different doses of colchicine in patients' peripheral blood mononuclear cell at week 30 with respect to baseline.

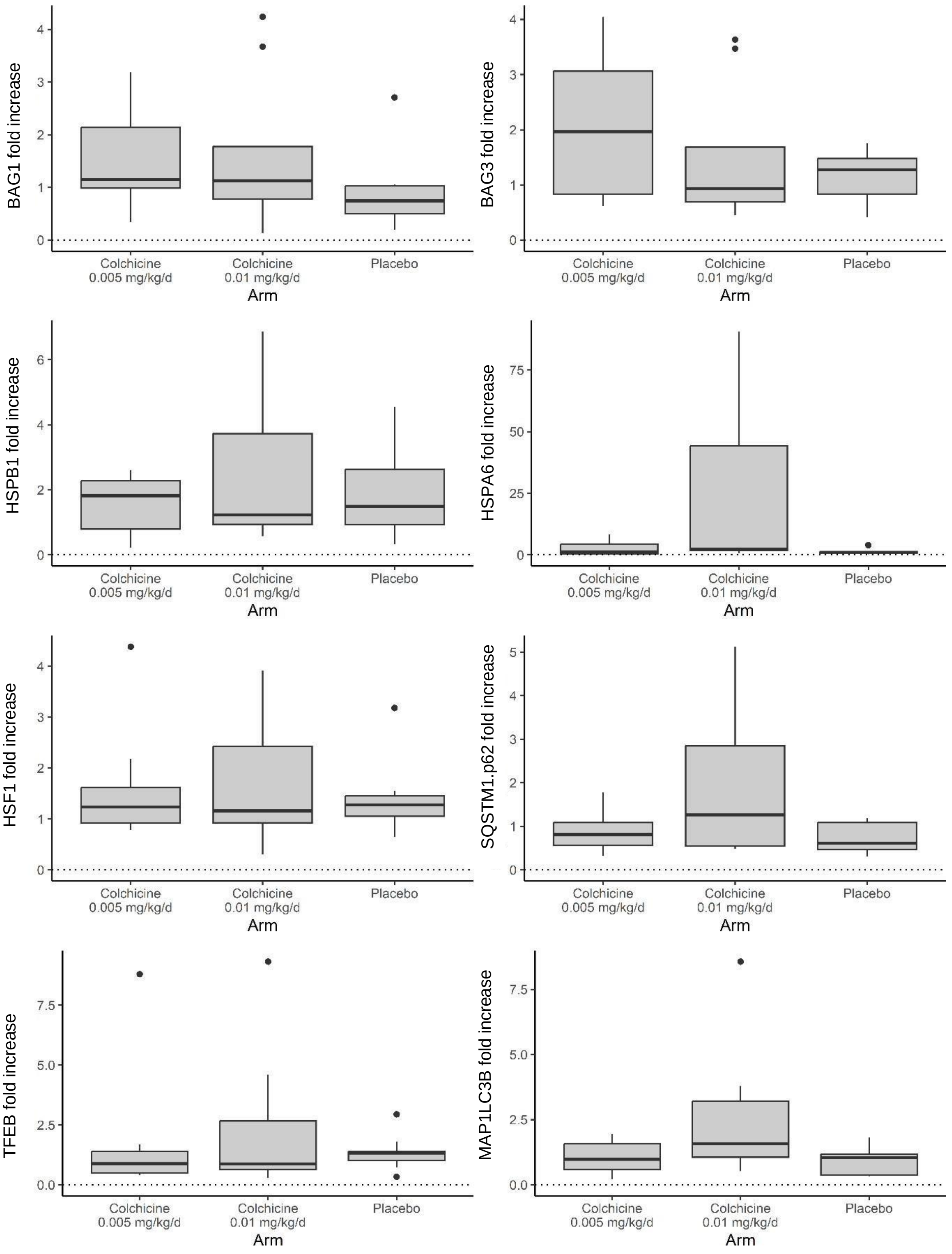

Supplementary Figure 6. Enrichment of defective ribosome products inside arsenite-induced stress granules in fibroblasts from ALS patients at baseline and treatment end.

Different color lines represent the enrichment of defective ribosome products (DRiPS) inside arsenite-induced stress granules (SGs) in fibroblasts from ALS patients at baseline (A) and at treatment end (B); DRiPs enrichment is shown on the horizontal axis and percentual of SGs is shown on the vertical axis. Enrichment below or equal to 1 corresponds to absence of DRiPs' enrichment inside SGs. Globally, 39 fibroblast lines from 39 ALS patients were generated before their enrolment in the study (A). Analysis of DRiPs' enrichment at baseline showed that the vast majority of SGs have a DRiPs' enrichment < 1.5 (as reported on the horizontal axis), but every line contains also SGs highly enriched in DRiPs (A). At week 30 only 15 fibroblast lines of 15 ALS patients could be examined, globally showing a higher percentage of SGs with DRiPs' enrichment > 2, although without differences across treatment arms and with highly variable distribution across fibroblast lines (B).

A

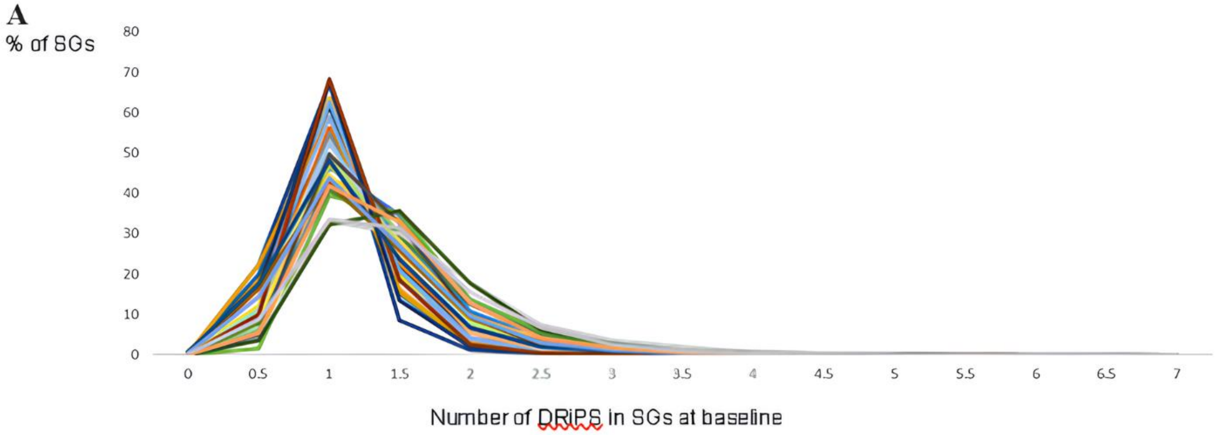

B

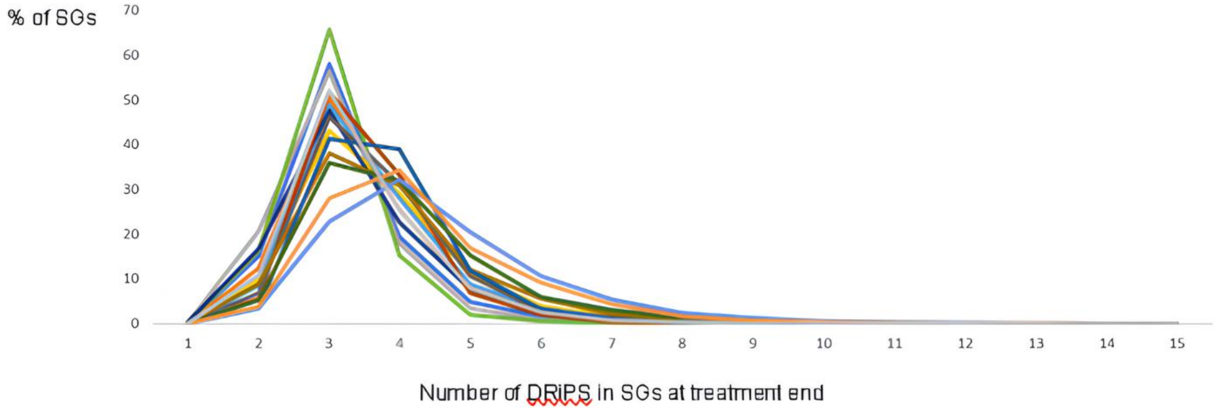

Supplementary Figure 7. Exosomes (EXOs) and microvescicles (MVs) production from patients' plasma across treatment arms at baseline (T0) and week 30 (T1).

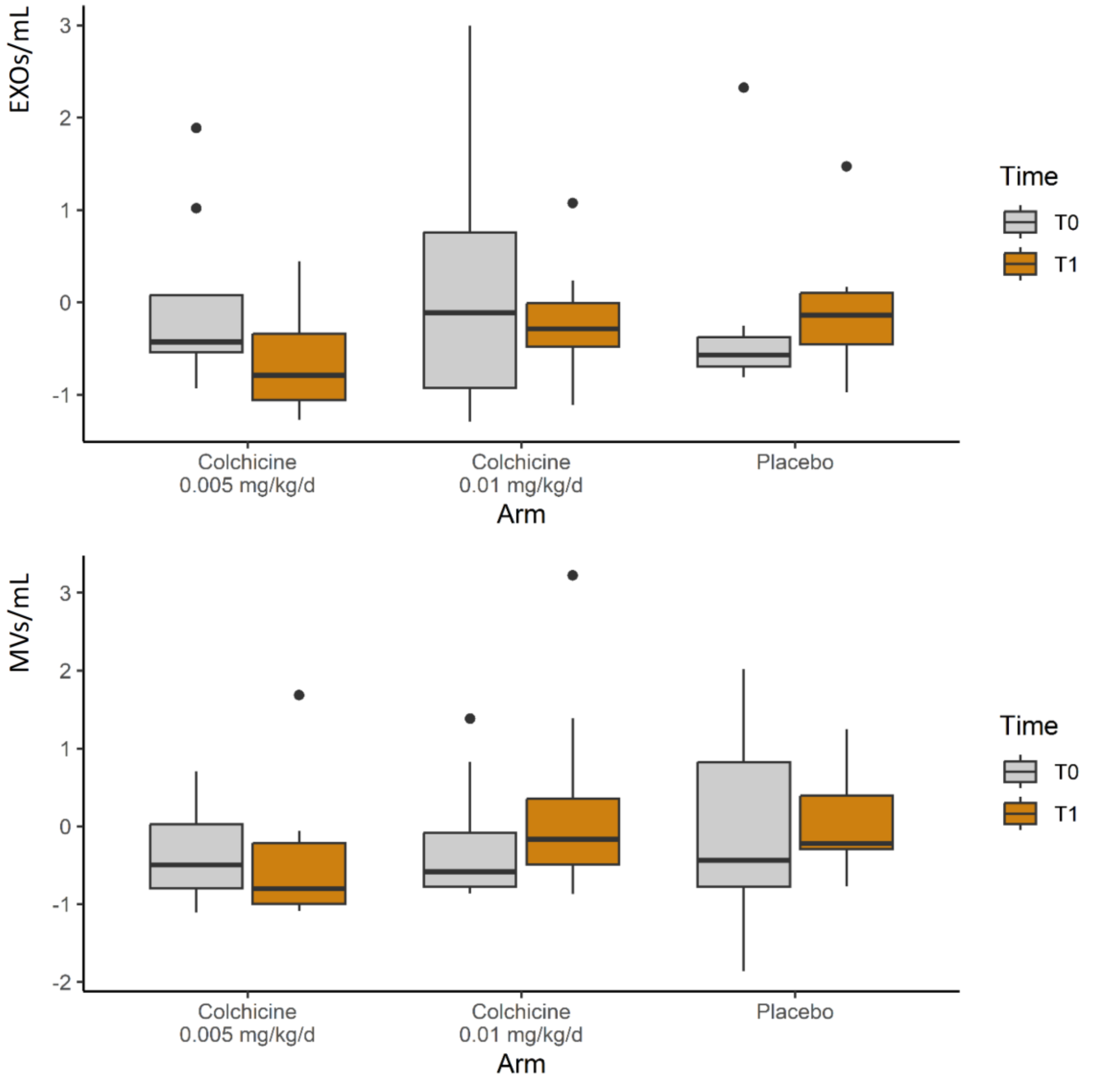

Supplementary Figure 8. Western Blot analysis in exosomes (EXOs) from patients' plasma measuring TDP- 43, TDP-35, and TDP- 25 expressions across treatment arms at baseline (T0), week 30 (T1) and week 54 (T2). Panel A shows western Blot analysis in exosomes (EXOs) from patients' plasma measuring TDP43, TDP-35, and TDP- 25 expressions across treatment arms at baseline (T0), week 30 (T1) and week 54 (T2). Panel B shows the relative full-size and uncropped gels.

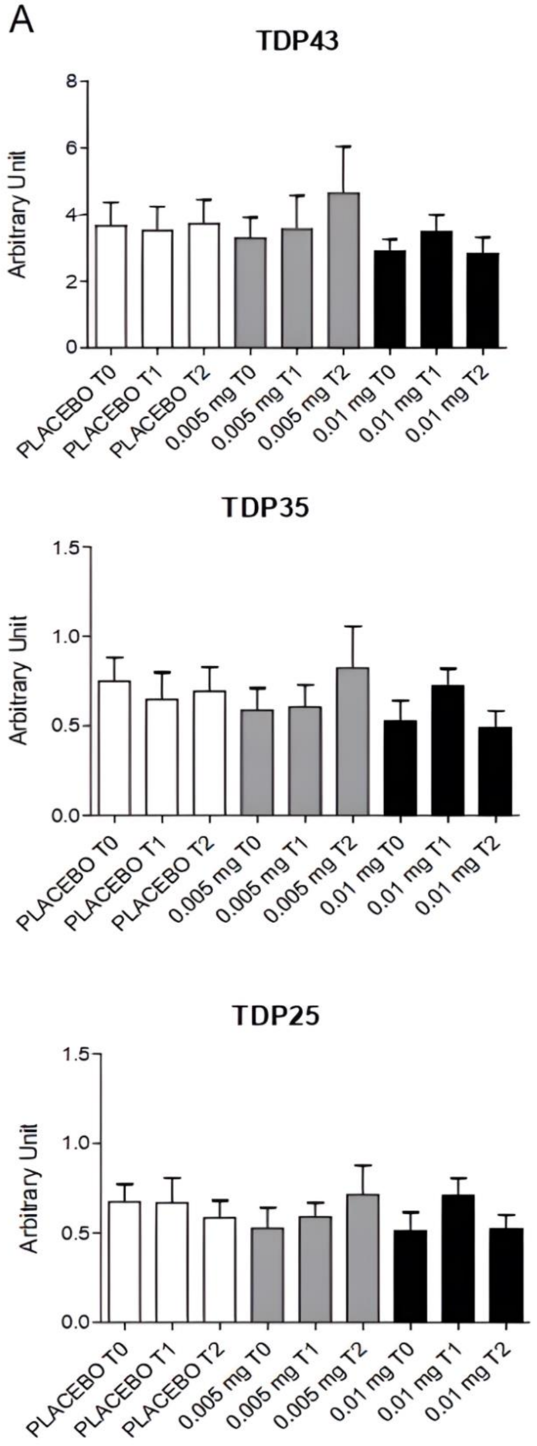

B

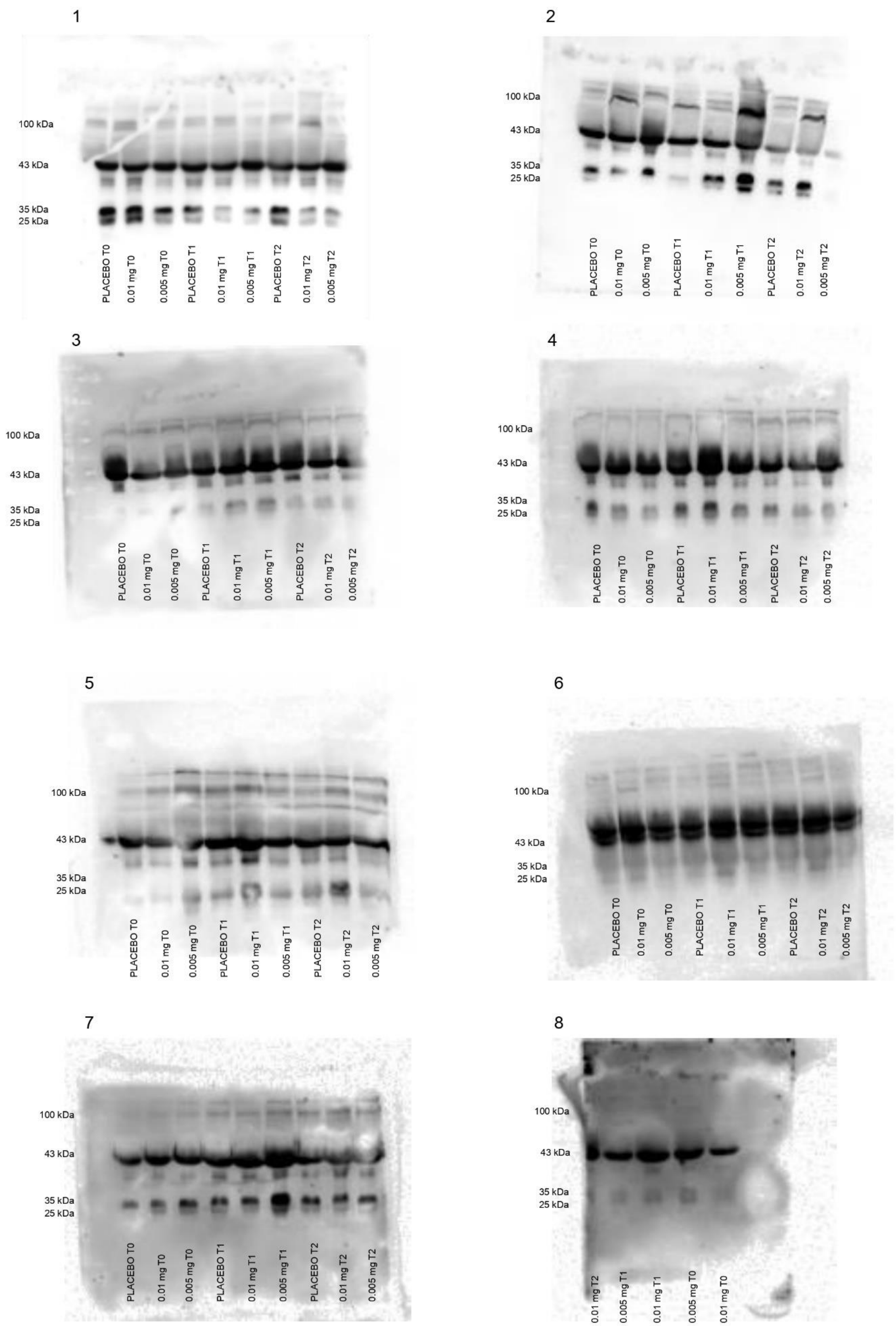
1
100 kDa
43 kDa
35 kDa
25 kDa
PLACEBO T0
0.01 mg T0
0.005 mg T0
PLACEBO T1
0.01 mg T1
0.005 mg T1
PLACEBO T2
0.01 mg T2
0.005 mg T2
2
100 kDa
43 kDa
35 kDa
25 kDa
PLACEBO T0
0.01 mg T0
0.005 mg T0
PLACEBO T1
0.01 mg T1
0.005 mg T1
PLACEBO T2
0.01 mg T2
0.005 mg T2
3
100 kDa
43 kDa
35 kDa
25 kDa
PLACEBO T0
0.01 mg T0
0.005 mg T0
PLACEBO T1
0.01 mg T1
0.005 mg T1
PLACEBO T2
0.01 mg T2
0.005 mg T2
4
100 kDa
43 kDa
35 kDa
25 kDa
PLACEBO T0
0.01 mg T0
0.005 mg T0
PLACEBO T1
0.01 mg T1
0.005 mg T1
PLACEBO T2
0.01 mg T2
0.005 mg T2
5
100 kDa
43 kDa
35 kDa
25 kDa
PLACEBO T0
0.01 mg T0
0.005 mg T0
PLACEBO T1
0.01 mg T1
0.005 mg T1
PLACEBO T2
0.01 mg T2
0.005 mg T2
6
100 kDa
43 kDa
35 kDa
25 kDa
PLACEBO T0
0.01 mg T0
0.005 mg T0
PLACEBO T1
0.01 mg T1
0.005 mg T1
PLACEBO T2
0.01 mg T2
0.005 mg T2
7
100 kDa
43 kDa
35 kDa
25 kDa
PLACEBO T0
0.01 mg T0
0.005 mg T0
PLACEBO T1
0.01 mg T1
0.005 mg T1
PLACEBO T2
0.01 mg T2
0.005 mg T2
8
100 kDa
43 kDa
35 kDa
25 kDa
0.01 mg T2
0.005 mg T1
0.01 mg T1
0.005 mg T0
0.01 mg T0

4. References

1. Crippa V, D'Agostino VG, Cristofani R, et al. Transcriptional induction of the heat shock protein B8 mediates the clearance of misfolded proteins responsible for motor neuron diseases. *Sci Rep*. 2016;6(1):22827. doi:10.1038/srep22827
2. Galbiati M, Meroni M, Boido M, et al. Bicalutamide and Trehalose Ameliorate Spinal and Bulbar Muscular Atrophy Pathology in Mice. *Neurotherapeutics*. 2023;20(2):524-545. doi:10.1007/s13311-023-01343-x
3. Casarotto E, Sproviero D, Corridori E, et al. Neurodegenerative Disease-Associated TDP-43 Fragments Are Extracellularly Secreted with CASA Complex Proteins. *Cells*. 2022;11(3):516. doi:10.3390/cells11030516
4. Seguin SJ, Morelli FF, Vinet J, et al. Inhibition of autophagy, lysosome and VCP function impairs stress granule assembly. *Cell Death Differ*. 2014;21(12):1838-1851. doi:10.1038/cdd.2014.103
5. Basso M, Pozzi S, Tortarolo M, et al. Mutant Copper-Zinc Superoxide Dismutase (SOD1) Induces Protein Secretion Pathway Alterations and Exosome Release in Astrocytes. *Journal of Biological Chemistry*. 2013;288(22):15699-15711. doi:10.1074/jbc.M112.425066
6. Thompson AG, Gray E, Mäger I, et al. CSF extracellular vesicle proteomics demonstrates altered protein homeostasis in amyotrophic lateral sclerosis. *Clin Proteomics*. 2020;17(1):31. doi:10.1186/s12014-020-09294-7

5. Abbreviations List

ADL	Activity Daily Living
AE	Adverse Event
AIFA	Agenzia Italiana del Farmaco
ALS	Amyotrophic lateral sclerosis
ALSAQ40	The 40 item Amyotrophic Lateral Sclerosis Assessment Questionnaire
ALSFRS-R	Amyotrophic Lateral Sclerosis Functional Rating Scale Revised
ATGs	Autophagy related genes
BCA	Bicinchoninic acid assay
BMI	Body Mass Index
cDNA	Complementary DNA
CK	Creatine kinase
CI	Confidence interval
CRF	Case Report Form
CSF	Cerebrospinal fluid
CYP3A4	Cytochrome P450 3A4
DRiPs	Defective Ribosomal Products
DMSO	Dimethylsulfoxide
ECLISSE	Euromed Clinical Supply Services S.R.L
EDTA	Ethylenediamine tetra acetic acid
EXO	Exosome
FBS	Fetal bovine serum
FRA	Filter Retardation Assay
FVC	Forced vital capacity
G3BP1	GTPase-activating protein-binding protein 1
GADPH	Glyceraldehyde-3-Phosphate Dehydrogenase
GCP	Good Clinical Practice
HCl	Hydrogen Chloride

HR	Hazard Ratio
HSC70	Heat-Shock Cognate 70
HSF1	Heat Shock Transcription Factor 1
HSPB8	Heat Shock Protein B8
HSPA6	Heat Shock Protein Family A (Hsp70) Member 6
ICH	International Council for Harmonization
IL	Interleukin
IMP	Investigational Medical Product
IQR	Interquartile range
ITT	Intent-to-treat
MAP1LC3B (or LC3)	Microtubule-Associated Proteins 1A/1B Light chain 3B
MCP1	Monocyte Chemoattractant Protein 1
MD	Mean Difference
MDR1	Multidrug resistance 1 gene
MedDRA	Medical Dictionary for Regulatory Activities
MN	Motor Neuron
MRC	Medical Research Council scale
mRNA	Messenger RNA
MV	Microvescicle
NF	Neurofilament
NfL	Neurofilament light chain
Op-PURO	O-Propargyl-puromycin
OPTN	Optineurin
OR	Odds ratio
PBMC	Peripheral blood mononuclear cell
pNfH	phosphorylated neurofilament heavy chain
P-gp	P-glycoprotein
PP	Per Protocol
RIPA	Radioimmunoprecipitation Assay

RR	Relative risk
RT-qPCR	Real Time quantitative Polymerase Chain Reaction
SAE	Severe Adverse Event
SD	Standard Deviation
SDS	Sodium Dodecyl Sulphate
SG	Stress granule
NSRI	Serotonin-norepinephrine reuptake inhibitor
SOD1	Superoxide dismutase 1
SSRI	Selective serotonin reuptake inhibitor
SQSTM1/p62	Sequestosome 1/protein 62
TDP43	Transactive response DNA binding protein 43 kDa
TFEB	Transcription Factor EB
UBQLN	Ubiquilin 2
WB	Western Blot
